# Supplementary material for: Observation of interband Berry phase in laser-driven crystals
Source: Nature. 2024 Jan 17;626(7997):66–71. doi: 10.1038/s41586-023-06828-5 (PMC10830408; doi:10.1038/s41586-023-06828-5)
Supplement: Supplementary file 1 — Supplementary Information [file 41586_2023_6828_MOESM1_ESM.pdf]

nature portfolio

https://doi.org/10.1038/s41586-023-06828-5

Supplementary information

Observation of interband Berry phase in laser-driven crystals

In the format provided by the authors and unedited

SI Guide

1 Experimental Setup **1**

2 α- quartz: structure, symmetries and DFT calculations **2**

3 Interband mechanism in α- quartz HHG **6**

3.1 First-principle simulations 6

3.2 Additional experimental measurements 9

3.3 Analysis of HHG mechanisms based on macroscopic simulations . . 16

3.3.1 Simulation Model 18

3.3.2 Resolving Intra- and Interband Emission in the Far-Field . 21

3.3.3 Macroscopic Simulation Results 23

3.4 Physical interpretation of the interplay between inter-band and intra-band mechanisms under different conditions 27

3.5 The physics of real and virtual excitations in the strong field regime and their relative importance in HHG 30

4 Semi classical trajectory analysis **32**

4.1 Single-color linearly-polarized light 34

4.2 Single-color elliptically-polarized light 36

4.3 Two-color cross-polarized light 38

5 HHG Spectroscopy in broken inversion crystals- Quantum analysis **40**

5.1 Interband emission 40

5.2 Saddle point approximation 42

5.3 Adding a parallel perturbation 44

5.4 Discrete Berry phase 48

5.5 Differences between Berry phase and Berry curvature 52

6 α–quartz HHG – quantum analysis **54**

6.1 Linearly Polarized Field 54

6.2 Elliptically Polarized Field 57

6.2.1 Reconstruction of the Berry Phase 58

6.2.2 Theoretical Berry phase calculation 59
6.3 Two-color Field 61
6.3.1 Theoretical study 64

1 Experimental Setup

The experiment was performed using a near-infrared laser source (Light Conversion TOPAS-HE) having a center wavelength of $\sim 1200nm$, at $1kHz$ repetition rate and $50fs - 70fs$ pulse duration. Figure 1 schematically describes the experimental set

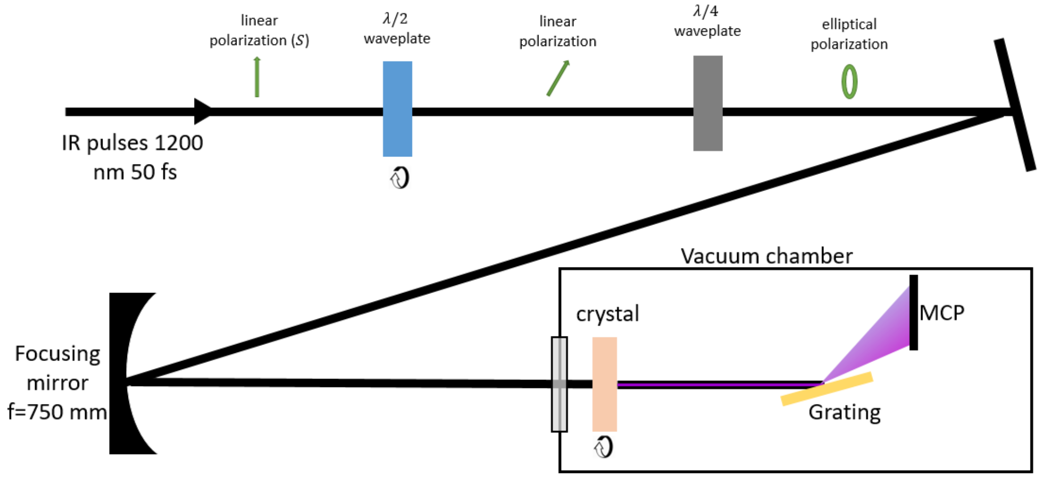

Figure 1: Schematic description of HHG spectroscopy setup, driven by an elliptically-polarized driving field.

up for HHG spectroscopy measurements, driven by an elliptically polarized light. The ellipticity of the laser field was controlled with a motorized half waveplate, followed by a quarter waveplate. This combination allowed us to scan the ellipticity degree of the light, while keeping the main axis of the ellipse fixed. The pulse energy, measured before the vacuum chamber was in the range of $300 - 400\mu J$ and the beam was focused using 75cm concave gold mirror. We estimated the laser field intensity to be in the range of $2 - 5 \cdot 10^{13} \frac{W}{cm^2}$. The beam was focused into a free-standing single crystal of z-cut ($< 100 >$) $20\mu m$ thick α-quartz sample (SiO2) placed at the imaging plane of an XUV spectrometer. The orientation of the crystal axes with respect to the laser polarization was controlled by a rotation motor.

Figure 2 schematically describes the second part of the experiment, two-color HHG spectroscopy. A weak second harmonic (SH) field was generated using a $100\mu m$ type-I phase barium borate (BBO) (BaB_2O_4) crystal, orthogonally polarized to the fundamental field's polarization. The intensity of the SH field was less

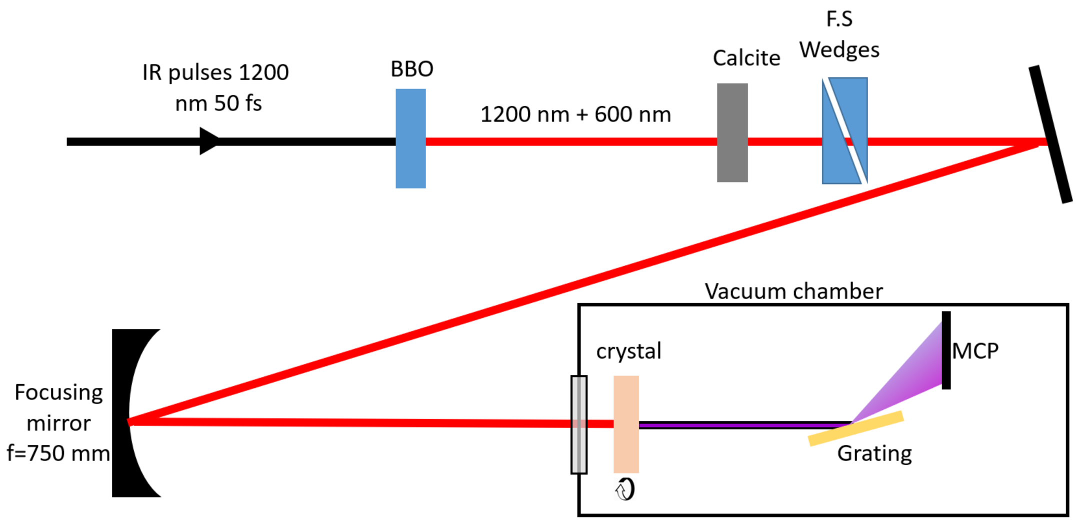

Figure 2: Two-color HHG spectroscopy setup

than 5% the fundamental intensity. A birefringent crystal (calcite) was used to compensate for group-velocity dispersion between the two fields, and their relative sub-cycle delay was controlled using a pair of fused silica wedges.

2 α- quartz: structure, symmetries and DFT calculations

α-quartz has a three fold rotational (C_3) symmetry around the c-axis, and a twofold symmetry (C_2) around the a-axis. Its space group is denoted as $P3_221$. For a laser field propagating in parallel to the c-axis, the HHG interband interaction can be described within the field's polarization plane, the c-plane. In the two-dimensional projection of quartz onto this plane, the C_2 symmetry of the crystal is equivalent to a mirror symmetry, as can be seen in the illustration in figure 3a. In addition, the atomic structure in the c-plane can be split into three identical patterns, repeated every 120°, thus demonstrating C_3 symmetry. Additionally, the lines connecting two S_i atoms through the center correspond to the C_2 axes. To describe the dynamics of the crystal's electronic eigenmodes, it is useful to examine these symmetries in the reciprocal crystal momentum space (k-space).

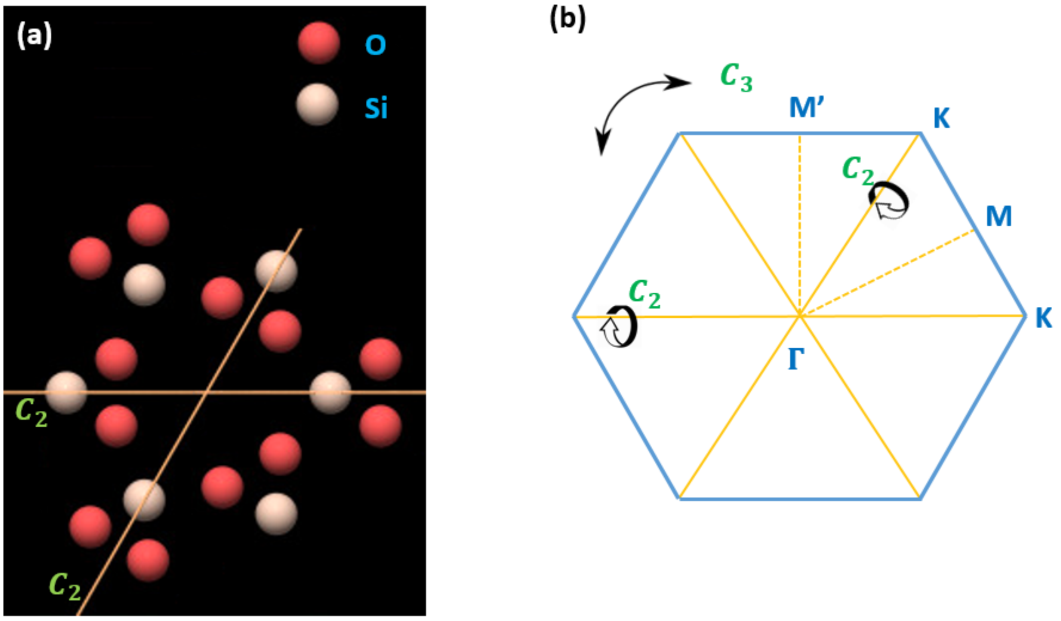

Figure 3: (a) An α-quartz projection of atoms onto the c-plane, demonstrating C_3 in-plane symmetry, and C_2 out-plane symmetry. The golden lines represent C_2 axes. (b) The first Brillouin zone of quartz, displaying the C_2 and C_3 symmetries in the reciprocal space. The solid golden lines correspond to the $\Gamma - K$ direction and the dashed lines show correspond to $\Gamma - M$ and $\Gamma - M'$.

Accordingly, figure 3b depicts the first Brillouin zone (BZ) of quartz, highlighting the main symmetry axes, $\Gamma - K$ and $\Gamma - M$, corresponding to the $0°$ and $30°$ rotational directions in the main text, respectively. Each $\Gamma - K$ line coincides with a C_2 symmetry axis. The BZ is symmetric with respect to a $120°$ rotation, thus respecting the C_3 symmetry as well.

The electronic structures were extracted by first principle calculations, performed in the frame work of density-functional theory by the Vienna Ab initio Simulation Package (VASP) [1, 2]. The electron exchange-correlation effect is described by the Perder-Burke-Ernzerhof (PBE) formulation, under the generalized gradient approximation (GGA)[3]. The projector augmented wave (PAW) pseudopotential [4, 5] was used to describe the interaction between electrons and nuclei. An energy cutoff as 520 eV was employed for the plane wave basis. The $10\times10\times10$ Γ-centered Monkhorst-Pack grids were employed to sample the Brillouin zone for the relaxation of all structures. The Wannier function-based tight-binding Hamil-

tonian of alpha-quartz is obtained by generating the maximally localized Wannier functions from the Wannier90 software [6], which is interfaced to VASP. The electronic structures and Berry phase related properties are calculated from the tight-binding-type Hamiltonian.

The band structure of quartz is displayed in figure 4a. The highest valence band (band 12, green curve around $0eV$) is separated from the lowest conduction band (band 13, light blue curve) by a large band gap, having a minimal value of $\sim 9.3eV$ around the Γ point. By closely examining the conduction bands, one can notice a few distinctive features of the lowest one. Compared with the other conduction bands, it spans over the largest energy range across the BZ, while retaining the largest band gap from all other conduction bands. These features allow us to study this band's k-space intrinsic properties as well as their mapping to energy via the emitted HHG.

Finally, we describe the intraband Berry curvature, defined as:

$$\boldsymbol{\Omega}_n(\boldsymbol{k}) = \nabla_{\boldsymbol{k}} \times \boldsymbol{\mathcal{A}}_n(\boldsymbol{k}), \; \boldsymbol{\mathcal{A}}_n(\boldsymbol{k}) = i \langle u_{n,\boldsymbol{k}}(\boldsymbol{x}) | \nabla_{\boldsymbol{k}} | u_{n,\boldsymbol{k}}(\boldsymbol{x}) \rangle \tag{2.1}$$

where $\boldsymbol{\mathcal{A}}_n$ is the Berry connection, and $|u_n\rangle$ is the periodic part of the Bloch wavefunction for band n. When a charge carrier is driven across k-space by an external laser field, the Berry curvature affects its intraband propagation within band n by introducing a lateral drift to its motion due to anomalous velocity, defined as:

$$\boldsymbol{v}_n(\boldsymbol{k}) = -\frac{e}{\hbar}\boldsymbol{F}(t) \times \boldsymbol{\Omega}_n(\boldsymbol{k}) \tag{2.2}$$

where $\boldsymbol{F}(t)$ is the driving field. The anomalous velocity mostly affects the evolution of the interaction in the polarization plane, due to phase matching considerations [7, 8]. For a field propagating along z, while being polarized predominantly along y, the interaction is mostly affected by the x component of $\boldsymbol{v}_n$. Therefore, Ω_n^z, i.e. the z component of the Berry curvature, is considered as the the main contribution affecting the intraband anomalous interaction.

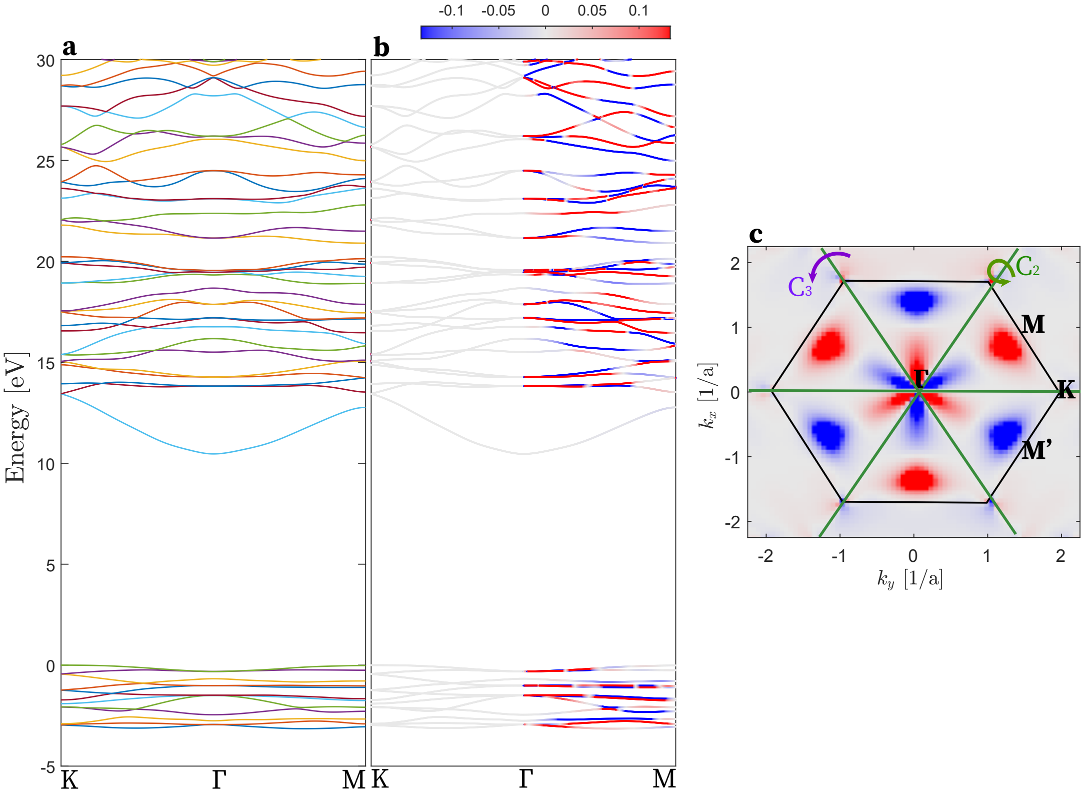

Figure 4: (a,b) α-quartz band structure along symmetry axes $K-\Gamma-M$ as extracted from DFT. In (a) each band is distinguished from neighboring bands by different colors. In (b) each band is color-coded with the band's Berry curvature's z component (parallel to field propagation direction) according to eq. 2.1 in main text (Berry curvature units are $[\frac{1}{a^2}]$ where a is the lattice constant). (c) The Berry curvature's z component of conduction band 14. The black dashed lines represent the edges of the first Brillouin zone. The golden lines represent C_2 axes.

The Berry curvature was calculated by DFT for each band and its z component is displayed in figure 4b along the $\Gamma-M$ and $\Gamma-K$ lines. As can be clearly observed, the Berry curvature can grow to large values along the $\Gamma-M$ symmetry axis. In contrast, along $\Gamma-K$ the Berry curvature is zero across all bands. This is a result of its anti-symmetric nature with respect to C_2 symmetry. The $2D$ Berry curvature is displayed in figure 4c for band 14, illustrating its angular dependence. Due to the C_2 symmetry, the Berry curvature flips its sign across the $\Gamma-K$ axis. In addition, due to the C_3 symmetry, the Berry curvature repeats itself after 120° rotation, with the Γ point acting as the center of rotation.

3 Interband mechanism in α- quartz HHG

The two major complementary mechanisms responsible for high harmonic generation in solids are associated with intraband currents [9–11] and interband polarization [12]. The balance between them depends on the laser parameters (carrier frequency, intensity and pulse duration), the properties of the medium, and can change across the harmonic spectrum.

We have performed an entirely new experimental study that resolves the origin of high harmonic emission in quartz in our experiments, showing the dominant role of the interband mechanism under our experimental conditions. In addition, we have performed systematic theoretical analysis , including full first-principles microscopic calculations in quartz and also simplified modeling of the macroscopic far-field response. Our theoretical results agree with different experimental observations [13–15] and show how the dominant harmonic generation mechanism switches for different experimental conditions. Below we provide detailed description of our experimental and theoretical study, starting with theory.

3.1 First-principle simulations

We performed time-dependent simulations in alpha quartz, using the code Quantum Espresso with a PBE functional on a 12x12x12 Monkhorst-Pack grid for computing the band structure. We then used the Wannier90 code [16] to generate the representation of the field-free Hamiltonian within the basis of maximally-localized Wannier functions. This procedure allows one to obtain consistent phase relation between the complex-valued dipole couplings at different crystal momenta. The Bloch states were projected onto the sp_3 orbitals of Si and the p orbitals of oxygen, totalling 30 bands. The α-quartz real-space, reciprocal space, and band structures are shown in Fig. 5. The Hamiltonian in the basis constructed from the Wannier functions was then propagated in the presence of the laser electric field using the density matrix formalism and the code we have described in ref. [17].

The laser wavelength was set to 1200nm, the full-width at half maximum pulse duration was set to 50fs, with the pulse intensity varied in the range of 0.9-30 TW/cm^2. The dephasing time was set to one or two cycles of the driving field,

$T_2 = T_0 = 2\pi/\omega$. Thanks to the shape of the Brillouin zone, the k_z component could be uncoupled in the calculation, allowing us to use fewer points along k_z and thus making the time-dependent calculations feasible within a reasonable amount of time.

We performed calculations for the carrier-envelope phase CEP=0 and CEP=π and averaged the results while imposing the symmetry constraints along each emission direction (currents are subtracted along $\Gamma - M$ and added along $\Gamma - K$). This does not affect symmetry-allowed harmonics, or the weights between intra- and inter-band components, which is our main interest, but removes the numerical artifacts that arise due to finite number of k-grid points, which can lead to weak symmetry-forbidden harmonic lines. With this procedure, convergence was obtained for a 100x100x10 k-point grid.

The current operator is defined as

$$\hat{\mathbf{J}} = \frac{i|e|}{\hbar} [\hat{\mathbf{r}}, \hat{H}(\mathbf{k}, t)], \tag{3.1}$$

where $\hat{\mathbf{r}}$ is the position operator and $\hat{H}(\mathbf{k}, t)$ is the time-dependent Hamiltonian. In the length gauge, $H(\mathbf{k}, t) = H_0(\mathbf{k}) + |e|\,\mathbf{E}(t)\cdot\mathbf{r}$, so that the interaction term commutes with the position operator and the current operator can be written as

$$\hat{\mathbf{J}} = \frac{i|e|}{\hbar} [\hat{\mathbf{r}}, \hat{H}_0(\mathbf{k})]. \tag{3.2}$$

Following the definition of the position operator given by Blount [18] in terms of the Berry connection $A_{nn'}$,

$$\hat{\mathbf{r}} f_n(\mathbf{k}) = i\partial_{\mathbf{k}} f_n(\mathbf{k}) + \sum_{n'} A_{nn'}(\mathbf{k}) f_{n'}(\mathbf{k}), \tag{3.3}$$

we can write the current operator in a way such that we can separate intraband and interband currents,

$$\hat{\mathbf{J}} = -\frac{|e|}{\hbar} \left(\nabla_{\mathbf{k}} H_{0,nm}(\mathbf{k}) - i \left[\mathbf{A}(\mathbf{k}), H_0(\mathbf{k})\right]_{nm} \right). \tag{3.4}$$

The first term is non-zero when $n = m$ and is associated with the intraband contribution, while the second term contains the interband contribution.

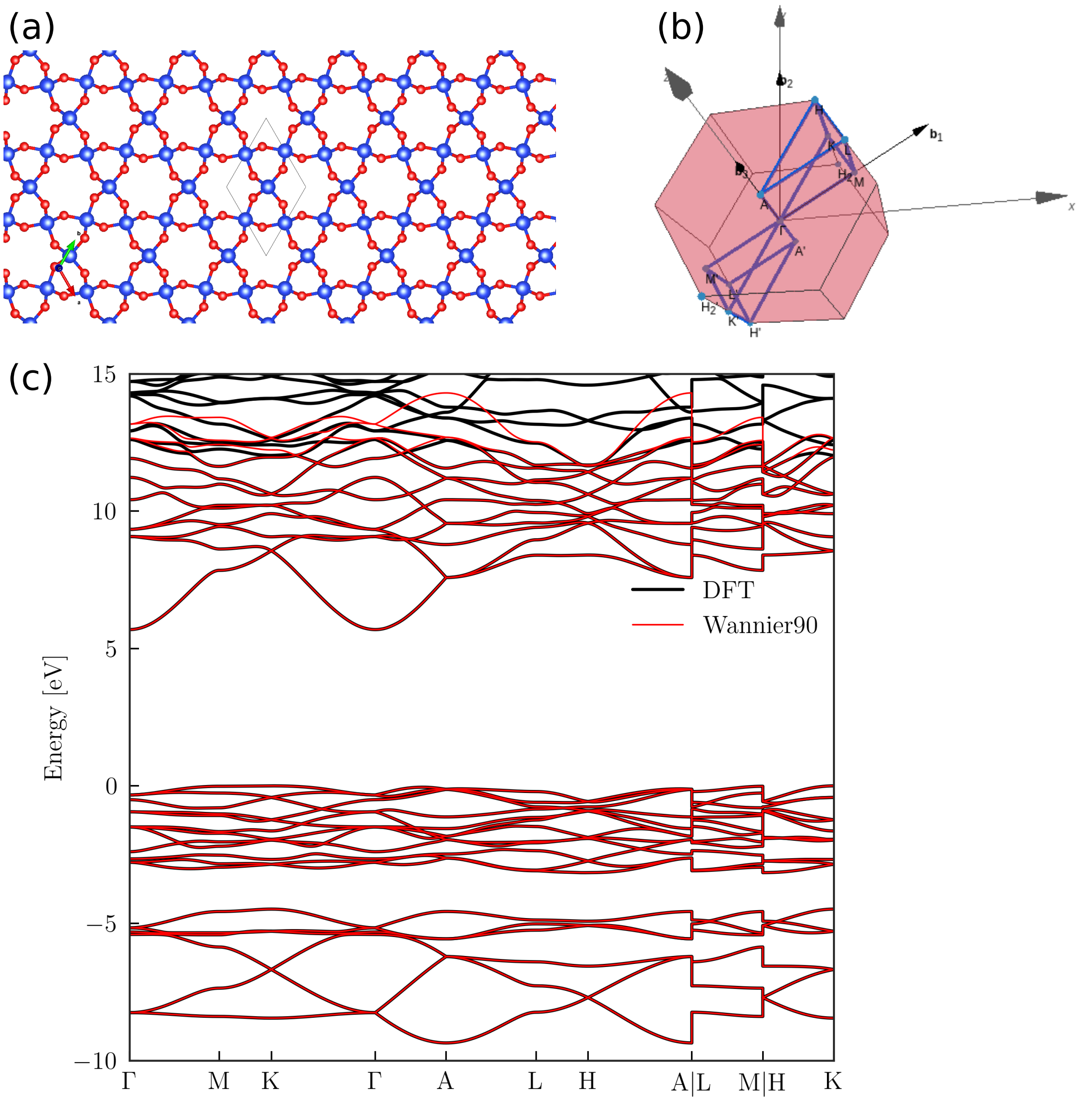

Figure 5: (a) α-quartz structure, (b) reciprocal space and (c) band structure. The unit cell is drawn in the center of the figure. The band structure was obtained with a PBE functional. Our Wannier90 interpolation projects over the p orbitals of the O atoms, and the sp_3 orbitals of the Si atoms. The HOMO energy is set to 0 eV. In the Wannierization procedure, we use a window up to 12 eV, ensuring optimal fit to the band structure withing this window.

Our results, presented in Figure 6, show that under the experimental conditions considered in our analysis, where the laser intensity approaches the experimental value, 30 TW/cm^2 the interband mechanism dominates. The same is found at lower intensities.

Figure 6 shows results for the field linearly polarized along the $\Gamma - M$ direction, i.e., along the direction where the Berry curvature is maximal. We see that the interband current contribution is higher by two to three orders of magnitudes (Figures 6c and 6d) compared with the intraband contribution. We note that these results do not depend on the dephasing time, as shown in Figure 7.

We shall see in the next chapters that these conclusions are consistent with available experiments for different laser parameters and by no means contradict the observations of intra-band mechanism at shorter wavelengths[13].

Our first-principle simulations predict that we should observe attochirp of high harmonics in our experimental conditions. This is the subject of our additional experimental measurements.

3.2 Additional experimental measurements

We have performed a systematic experimental study that reveals the underlying HHG mechanism in our experiments. This experiment aims to confirm the attochirp of the emitted harmonics in the spectral region of interest. To this end, we probe the HHG mechanism in quartz via the application of the in-situ scheme [19]. The in-situ scheme has been, developed during the past two decades, and applied in a large variety of atomic and molecular systems to resolve a range of fundamental phenomena such as the tunneling dynamics[20, 21] or multielectron processes[22].

The in-situ scheme has now been extended to condensed matter systems. This scheme was first applied by the NRC group to reveal the role of the interband mechanism in a low band gap crystal such as ZnO[12]. Later, this scheme was applied by several groups to identify the interband process in a high bandgap system such as MgO[23, 24]. This observation was then confirmed by alternative spectroscopic approaches such as the CEP scans[15, 25] or the appearance of spectral caustics[23]. Recently, the Weizmann and the MBI teams have applied the in-situ

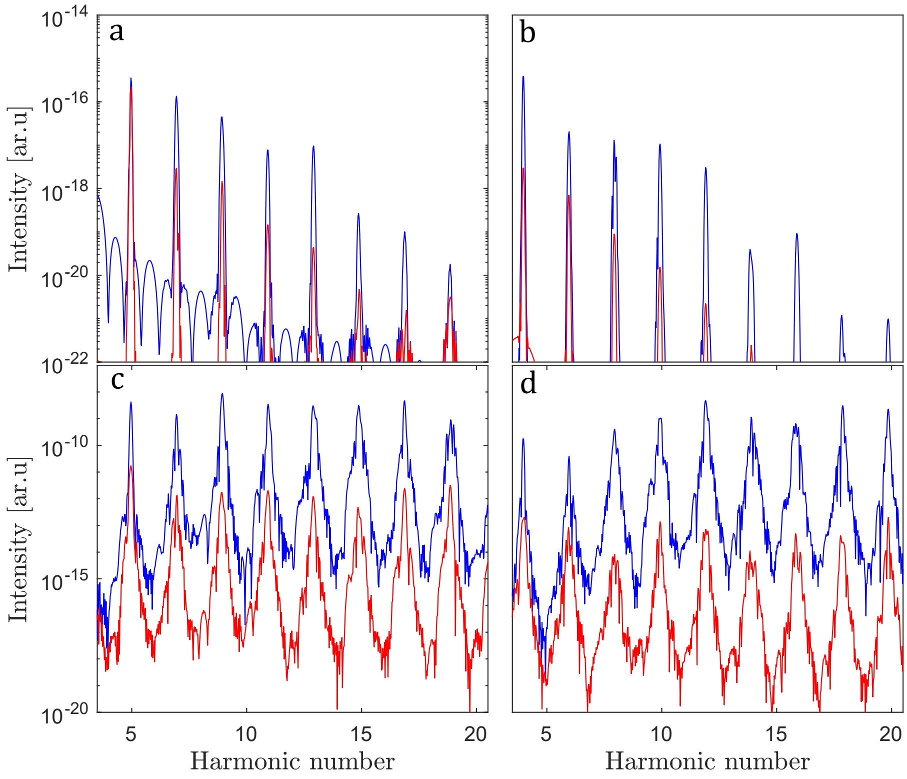

Figure 6: Calculated HHG spectrum for quartz along $\Gamma - M$, for the parallel (a,c) and perpendicular (b,d) components. The laser intensity is $I = 0.9$ TW/cm^2 in panels (a,b) and $I = 30$ TW/cm^2 in panels (c,d); the dephasing time is $T_2 = 2T_0$. The intra/interband currents are plotted in red/blue.

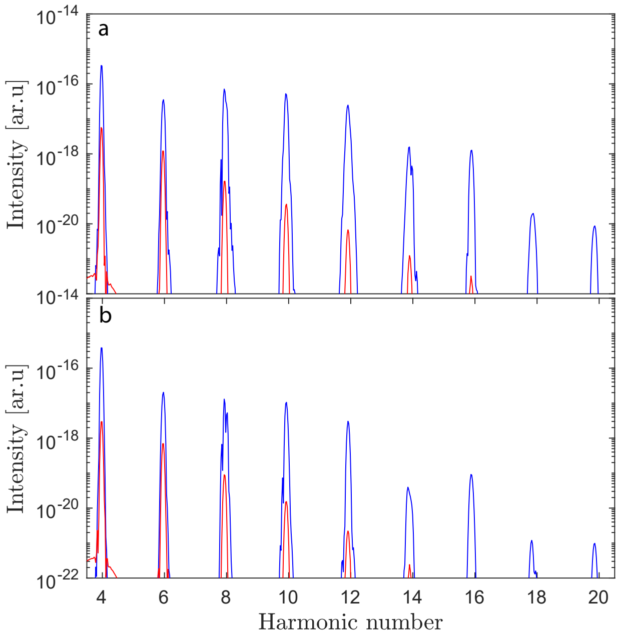

Figure 7: Comparison of the calculated HHG intensities for the perpendicular polarization component, for a laser intensity of $I = 0.9$ TW/cm^2, for $T_2 = T_0$ (a) and $T_2 = 2T_0$ (b).

scheme in MgO, and resolved laser-induced closing of the band-gap between adjacent conduction bands, revealing the mapping between the HHG and the different conduction bands[26].

Here, we apply the in-situ scheme in quartz. Our experiment consists of a strong IR field together with a weak second harmonic (SH) field, with a parallel polarization, controlling their sub-cycle delay τ. Each trajectory acquires an additional complex phase $\sigma(\tau)$, which is accumulated along the entire trajectory, serving as a sensitive label of its temporal properties. We can describe this phase shift as a perturbation to the total action accumulated by the electron as: $S(\tau) = S_0 + \sigma(\tau)$. The real part, $Re(\sigma)$, is associated with an additional phase accumulated by the electron after injection (tunnelling), while the imaginary part, $Im(\sigma)$, is associated with a small perturbation of the injection (tunneling) process[20]. The additional phase is mapped into the harmonics intensity of both even and odd harmonics.

Scanning τ modifies $\sigma(\tau)$ in a periodic manner, modulating the harmonic spectrum. These modulations (i) reveal the symmetry of the solid-state medium and

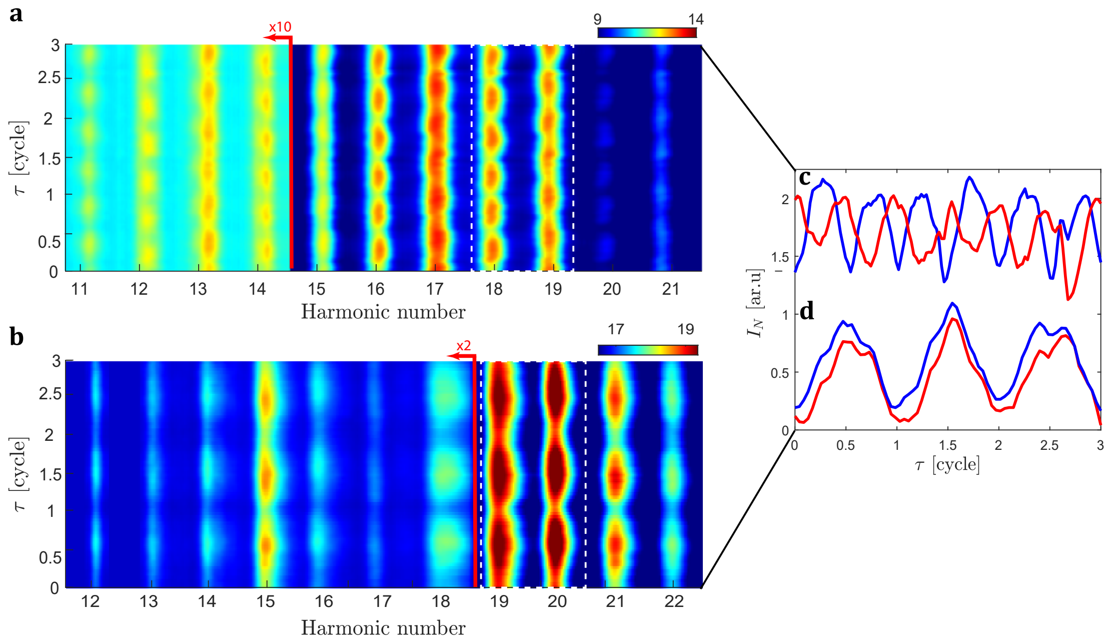

Figure 8: **Two-color HHG spectroscopy resolved in MgO and quartz crystals**. (a) and (b) Oscillating harmonic spectrum resolved in MgO and quartz crystals, respectively, as a function of the time delay between the two fields (presented with respect to the SH cycle period). (c) Comparison between the oscillation of the even (H18, red) and odd (H19, blue) harmonics, generated in MgO crystal, showing *out of phase* oscillation. (d) Comparison between the oscillation of the even (H20, red) and odd (H19, blue) harmonics, generated in quartz crystal, showing *in phase* oscillation.

(ii) provide insight into the dynamical properties of the interaction.

First, we compare the in-situ measurement performed in MgO and quartz. Figure 8 presents the HHG spectrum as a function of τ for MgO and quartz. Clear modulations of the harmonic signal are observed for both systems. Before we carefully analyze the phase associated with each harmonic order, we focus on two main observables: the modulation's periodicity and the modulation's phase of both even and odd harmonics. In MgO the harmonics oscillate with $2\omega_{SH}$ frequency (ω_{SH} is the SH frequency), where even and odd harmonics are modulated out of phase. In strong contrast, in quartz, the signal oscillates with ω_{SH} frequency, where odd and even harmonics are modulated approximately in phase. What is the origin of this striking difference between the two systems?

We can understand this difference by considering the generation process induced during two consecutive half cycles. The exact expressions are derived in the quantum analysis, in chapter 5.3, here we present a general description that cap-

tures the complex nature of the perturbation. The harmonic fields emitted during each half cycle can be described as: $E_{1,2} = \alpha_{1,2} e^{\varphi_{1,2}}$, where 1,2 represents the first and second half-cycles respectively. $\alpha_{1,2}$ represents the amplitude of the harmonic emission, while $\varphi_{1,2}$ represents its phase, which is dictated by the semi-classical action, S, as well as the dipole phase. Adding the SH field adds a complex perturbation to the harmonic's field emitted during the two half-cycles, represented by σ_1 and σ_2. The HHG spectrum encodes the interference between the two emission bursts according to:

$$I^N \propto |\alpha_1 e^{i\varphi_1} e^{i\sigma_1(\tau)} \pm \alpha_2 e^{i\varphi_2} e^{i\sigma_2(\tau)}|^2 \tag{3.5}$$

where + is for odd and − is for even harmonics. In systems with inversion symmetry [19, 20]: $\alpha_1 = \alpha_2$, $\varphi_1 = \varphi_2$, and $\sigma_1 = -\sigma_2$, therefore:

$$I^N \propto |\alpha|^2 |e^{i\sigma_1(\tau)} \pm e^{i\sigma_2(\tau)}|^2 \propto \begin{cases} 1 + (\sigma^i)^2 - (\sigma^r)^2 & N \text{ is odd} \\ (\sigma^i)^2 + (\sigma^r)^2 & N \text{ is even} \end{cases} \tag{3.6}$$

Here $\sigma^i_{1,2} = Im(\sigma_{1,2})$ and $\sigma^r_{1,2} = Re(\sigma_{1,2})$.

Thus, in inversion symmetric systems, the first order vanishes and the perturbation appears in the **second** order only. Scanning the two-color delay thus modulates σ by ω_{SH} and the harmonic signal by $2\omega_{SH}$ oscillations. In contrast, in systems with broken inversion symmetry the response to the perturbation is completely different. In this case, the harmonic signal behaves as:

$$I^N \propto \begin{cases} 2\alpha_1\alpha_2 \cos\Delta\varphi(1 + \sigma_1^i + \sigma_2^i) + 2|\alpha_1|^2\sigma_1^i + 2|\alpha_2|^2\sigma_2^i - 2\alpha_1\alpha_2 \sin\Delta\varphi(\sigma_1^r - \sigma_2^r) + O(\sigma^2) & N \text{ is odd} \\ -2\alpha_1\alpha_2 \cos\Delta\varphi(1 + \sigma_1^i + \sigma_2^i) + 2|\alpha_1|^2\sigma_1^i + 2|\alpha_2|^2\sigma_2^i + 2\alpha_1\alpha_2 \sin\Delta\varphi(\sigma_1^r - \sigma_2^r) + O(\sigma^2) & N \text{ is even} \end{cases} \tag{3.7}$$

where $\Delta\phi = \phi_1 - \phi_2$. Note that $\sigma(\tau)$ is a periodic function of the two-color delay τ. We see that the perturbation appears in the **first** order, leading to ω_{SH} oscillations.

This analysis fully captures our experimental observations of the different oscillation period in MgO crystal vs. quartz crystal.

Next, we note that when the perturbation is dominated by its real component, the neighboring even and odd harmonics oscillate out of phase. In contrast, when the perturbation is dominated by the imaginary component, the odd and even

harmonics will oscillate in phase[12, 20].

Clearly, the perturbation in MgO is dominated by the real component. In contrast, our experimental results reveal that the perturbation in quartz is dominated by its imaginary component. The dominant role of the imaginary perturbation originates from two important properties. The first is the high band gap of quartz ($9.3eV$), leading to crucial contribution of tunneling and therefore large imaginary action. The second is the asymmetry in tunneling during the two consecutive half cycles, associated with the symmetry breaking ($\sigma_1 \neq \sigma_2$) within each cycle.

Importantly, the tunneling step is common for both HHG mechanisms, inter-band and intra-band. The modulation associated with this step does not distinguish between the two. The interplay of inter-band and intra-band mechanisms is encoded in the real perturbation associated with the phase accumulated by the trajectory. For the inter-band mechanism, this phase reflects the length of the trajectories and the mapping between their length and the harmonic orders.

We can isolate the oscillating real perturbation from the imaginary one by combining the pairs of neighboring harmonic orders:

$$I^{N_{odd}} - I^{N_{even}} \propto \alpha_1 \alpha_2 \sin(\Delta\varphi)(\sigma_1^r - \sigma_2^r) \quad (3.8)$$

Such analysis removes the imaginary contribution and isolates the real perturbation.

Figure 9-12 present the modulation phase, focusing on the harmonic emission associated with the first conduction band (HH11-HH17), for the three values of the fundamental field intensity. We extract the modulation phases by performing Fourier analysis, resolving the phase of the Fourier peak at $2\omega_0$ [20, 27, 28]. The corresponding oscillating harmonic signals are presented in figures 10-12. We evaluate the phase error by performing a careful analysis in the Fourier domain. Such analysis extracts the phase error due to a random, spectrally flat noise, having an amplitude dictated by the SNR level in the Fourier domain. This analysis shows the modulation phase changes with the harmonic number, where the even and the odd harmonics are slightly out of phase. In addition, the phase variation of both even and odd harmonics strongly depends on the fundamental field intensity, becoming almost flat at high laser intensities.

In the next stage, we extract $I^{N_{odd}} - I^{N_{even}}$ and resolve the real part of the perturbation, encoded in the modulation phase of each pair of neighboring harmonics. Such analysis reveals the temporal information recorded in the experimental results (equation 3.8). Crucially, we find that the modulation phase of the real perturbation changes significantly with the harmonic order, resolving the clear fingerprint of the interband mechanism. The variation of this phase from one harmonic pair to another originates from the recombination dynamics and identifies the mapping between the recombination time and harmonic order. Such response has been well established in gas phase[19] and extended to condensed matter systems by Vampa et al in ZnO[12].

Our study captures the dependence of the time-energy mapping with the fundamental field's intensity. In our measurements, the spectral slope of the modulation phase decreases as we increase the fundamental field's intensity. Such a response is in full agreement with the semi classical inter-band picture. As we increase the laser intensity, the mapping between recombination time and harmonic order becomes more flat, and thus the attochirp reduces.

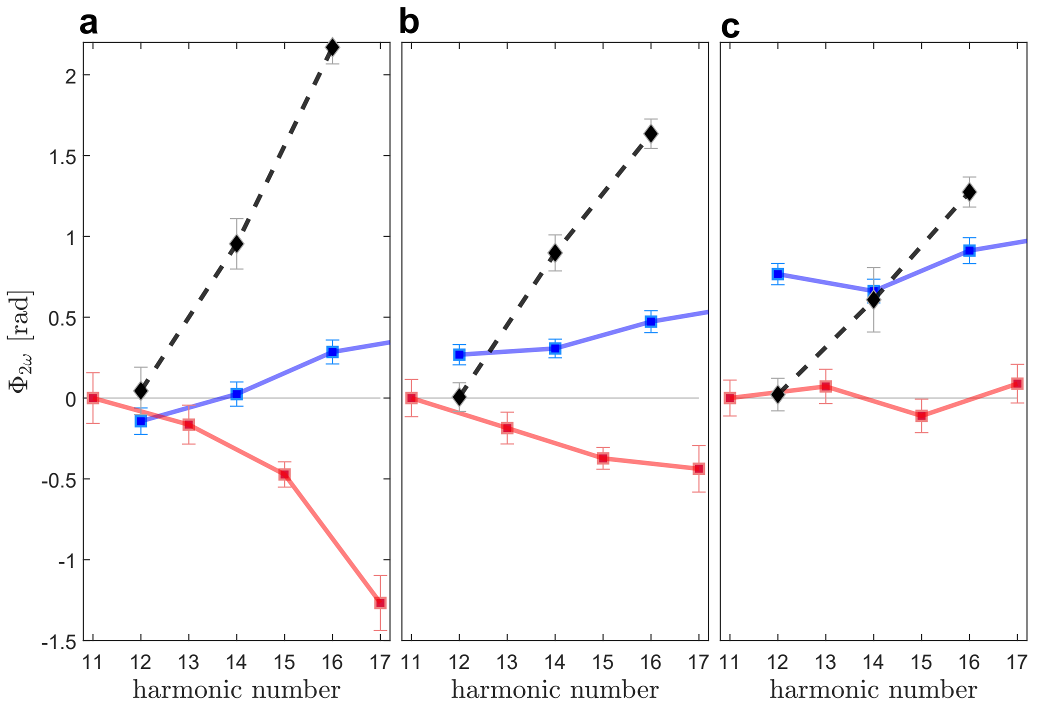

Figure 9: Identifying the interband mechanism captured by the harmonics' oscillation phase. (a-c) The oscillation phase of the even (blue) and the odd (red) harmonics, as well as the oscillation of their difference ($I^N_{odd} - I^N_{even}$, black), generated using $300\mu J$, $350\mu J$ and $400\mu J$ driving fields. The oscillation phase is extracted using Fourier analysis.

3.3 Analysis of HHG mechanisms based on macroscopic simulations

We now complement our microscopic analysis with macroscopic simulations, albeit for a simplified microscopic model. This additional allows us to better connect our numerical calculations with experimental results, since the latter deal with the far-field HHG signal. Crucially, this also allows us to better examine the interplay between the different HHG mechanisms under different experimental conditions, providing a broader picture and connecting to previous experimental works of HHG in quartz, performed under different experimental conditions.

To this end, we have developed a novel approach based on real-space-resolved evaluation of the generated light in the basis of Wannier orbitals.

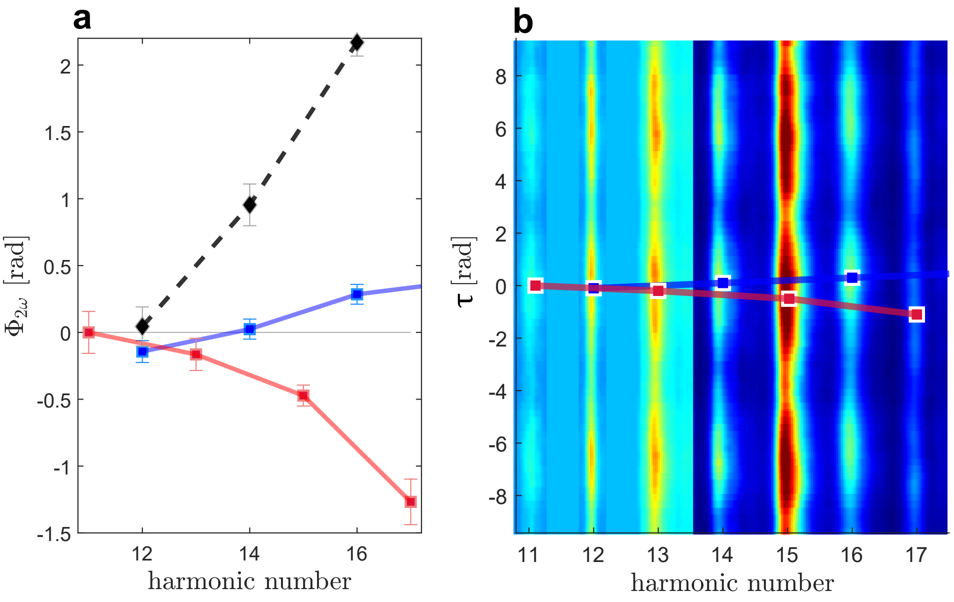

Figure 10: a, The extracted oscillation phase, using Fourier analysis, of the even (blue) and the odd (red) harmonics, as well as the oscillation of their difference ($I^N_{odd} - I^N_{even}$, black), generated using 300μJ driving field. b, The 2D HHG spectrum as a function of the two colors delay, together with the phase analysis presented in (a)

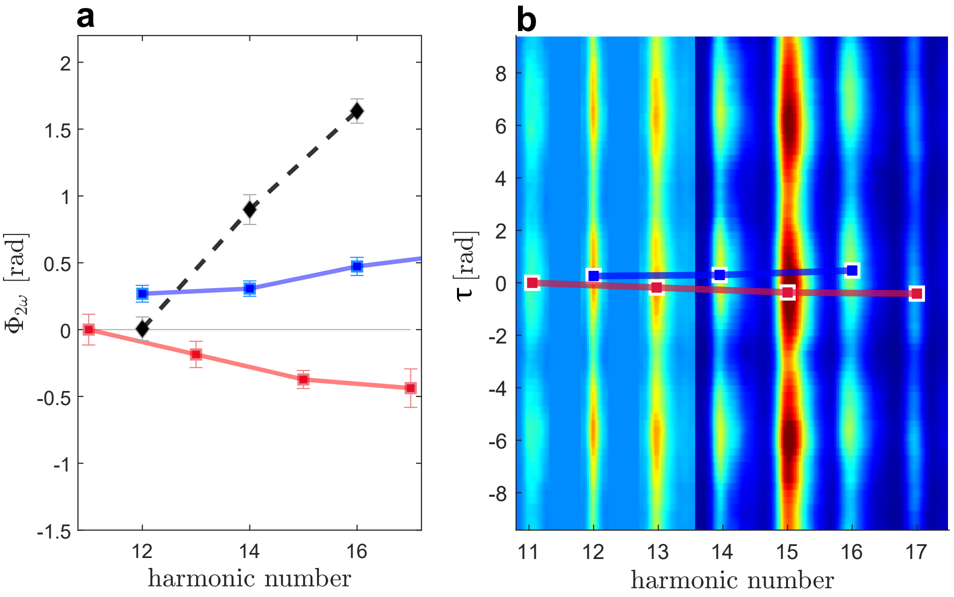

Figure 11: a, The extracted oscillation phase, using Fourier analysis, of the even (blue) and the odd (red) harmonics, as well as the oscillation of their difference ($I^N_{odd} - I^N_{even}$, black), generated using 350μJ drivng field. b, The 2D HHG spectrum as a function of the two colors delay, together with the phase analysis presented in (a).

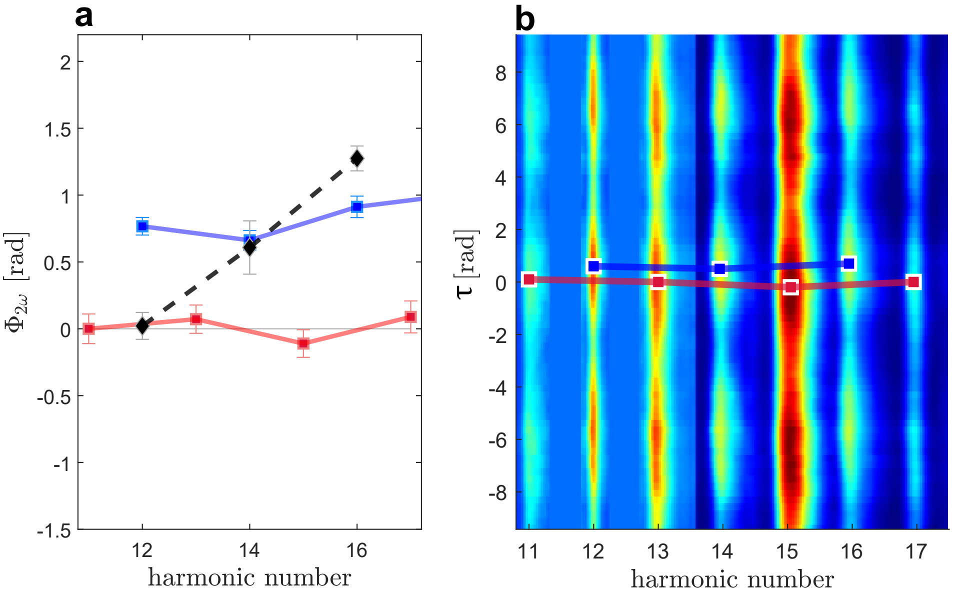

Figure 12: The extracted oscillation phase, using Fourier analysis, of the even (blue) and the odd (red) harmonics, as well as the oscillation of their difference ($I^N_{odd} - I^N_{even}$, black), generated using $400\mu J$ driving field. b, The 2D HHG spectrum as a function of the two colors delay, together with the phase analysis presented in (a).

3.3.1 Simulation Model

We begin by describing the simulation model, which is formulated in the Wannier basis. We consider a one-dimensional solid-state system with lattice constant $a_0 = 6$ a.u. described by a Mathieu-type potential with strength $v_0 = 0.75$ a.u. [29]. From the Bloch theorem, the eigenstates $|\psi_{m,k}\rangle$, of the Hamiltonian $\hat{H}_0$ of the system satisfy the following:

$$\hat{H}_0|\psi_{m,k}\rangle = \epsilon^k_m|\psi_{m,k}\rangle, \tag{3.9}$$

$$\langle x|\psi_{m,k}\rangle = e^{ikx}u_{m,k}(x), \tag{3.10}$$

$$p^k_{m,m'} = \langle\psi_{m,k}|\hat{p}|\psi_{m',k}\rangle, \tag{3.11}$$

where m denotes the band index, k is the crystal momentum, $\epsilon_m(k)$ is the energy of the state in band m with crystal momentum k, the Bloch function $u_{m,k}(x)$ reflects the periodicity of the lattice, and the momentum operator $\hat{p}$ is diagonal with respect to the crystal momentum. With these parameters, the system exhibits a

minimum band gap of 9.3 eV.

The representation of the system in the Wannier basis is obtained through a unitary transformation which relates the Wannier state in band m at lattice site R, $|\phi_{m,R}\rangle$, and the Bloch states in band m as follows [30]:

$$|\phi_{m,R}\rangle = \frac{1}{\sqrt{N}} \sum_k e^{-ikR} |\psi_{m,k}\rangle, \tag{3.12}$$

$$|\psi_{m,k}\rangle = \frac{1}{\sqrt{N}} \sum_R e^{ikR} |\phi_{m,R}\rangle, \tag{3.13}$$

where N denotes the number of considered states (i.e. k-points). From Eqs. (3.9-3.13), the field-free Hamiltonian $\hat{H}_0^{(W)}$ and momentum operator $\hat{p}^{(W)}$ in the Wannier basis are given as follows [31]:

$$\hat{H}_0^{(W)} = \sum_{\Delta_R} \hat{\mathcal{H}}_{\Delta_R}, \tag{3.14}$$

$$\hat{\mathcal{H}}_{\Delta_R} = \sum_m \tilde{\epsilon}_m^{\Delta_R} \sum_R |\phi_{m,R}\rangle\langle\phi_{m,R+\Delta_R}|, \tag{3.15}$$

$$\hat{p}^{(W)} = \sum_{\Delta_R} \hat{\mathcal{P}}_{\Delta_R}, \tag{3.16}$$

$$\hat{\mathcal{P}}_{\Delta_R} = \sum_{m,m'} \tilde{p}_{m,m'}^{\Delta_R} \sum_R |\phi_{m,R}\rangle\langle\phi_{m',R+\Delta_R}|, \tag{3.17}$$

where Δ_R denotes the separation between two Wannier states and

$$\tilde{\epsilon}_m^{\Delta_R} = \frac{1}{N} \sum_k \epsilon_m^k e^{-ik\Delta_R}, \tag{3.18}$$

$$\tilde{p}_{m,m'}^{\Delta_R} = \frac{1}{N} \sum_k p_{m,m'}^k e^{-ik\Delta_R}. \tag{3.19}$$

By expressing $\hat{p}$ as a summation over Δ_R, HHG emission can be resolved according to the separation between coherences.

We use the density matrix formalism using the velocity gauge to propagate the system in time and calculate HHG spectra [29, 31] at each radial position r along

the driving field beamfront in the focal plane. For all considered wavelengths, the driving field is a Gaussian beam with peak amplitude F_0, beam waist σ, carrier frequency ω_0, and pulse duration τ_0 of eight optical cycles:

$$F(r,t) = F_0 e^{-(r/\sigma)^2} e^{-(2t/\tau_0)^2} \cos(\omega_0 t). \tag{3.20}$$

The dipole current at radial position r of the driving field beamfront resolved by lattice site separation is calculated as the trace of the momentum operator in Eq. (3.17) and the time-dependent density matrix $\hat{\rho}(r,t)$ for each lattice site separation Δ_R, such that the time-dependent current $j_{\Delta_R}(r,t)$ and dipole emission spectrum $\tilde{j}_{\Delta_R}(r,\Omega)$ for separation Δ_R is given as follows:

$$j_{\Delta_R}(r,t) = \mathrm{Tr}\left[\hat{\mathcal{P}}_{\Delta_R}\hat{\rho}(r,t)\right], \tag{3.21}$$

$$\tilde{j}_{\Delta_R}(r,\Omega) = \int_{-\infty}^{\infty} j_{\Delta_R}(r,t) f(t) e^{i\Omega t} dt, \tag{3.22}$$

Where $f(t)$ is a Hann window function used for the Fourier transform. The total current and emission spectrum is obtained by summing Eqs. (3.21) and (3.22) for all Δ_R.

We then propagate the HHG emission into the far-field a distance z by first calculating the spectral beamfront in reciprocal space at radial momentum k_r and photon energy, $\tilde{\mathcal{J}}_{\Delta_R}(k_r,\Omega)$, as follows [32]:

$$\tilde{\mathcal{J}}_{\Delta_R}(k_r,\Omega) = 2\pi \int_0^{\infty} \tilde{j}_{\Delta_R}(r,\Omega) J_0(k_r r)\, r dr, \tag{3.23}$$

where $J_0(k_r r)$ is the zeroth-order Bessel function of the first-kind. We then define the far-field propagation kernel $U_\Omega(z)$ for frequency Ω and propagation distance z as

$$U_\Omega(z) = e^{i\bar{k}_\Omega z}, \tag{3.24}$$

$$\bar{k}_\Omega = \sqrt{k_\Omega^2 - k_r^2}, \tag{3.25}$$

where $k_\Omega = \Omega/c$ and c is the speed of light. The far-field spectral beamfront at radial

position r', $\tilde{j}^{(f)}(r', z, \Omega)$ is calculated by taking the inverse Hankel transform of the product of the reciprocal space spectral beamfront and the far-field propagation kernel for each frequency Ω:

$$\tilde{j}^{(f)}_{\Delta_R}(r', z, \Omega) = 2\pi \int_0^\infty U_\Omega(z) \tilde{\mathcal{J}}_{\Delta_R}(k_r, \Omega)\, J_0(k_r r')\, k_r dk_r. \tag{3.26}$$

3.3.2 Resolving Intra- and Interband Emission in the Far-Field

We resolve between intra- and interband HHG emission in two ways. For the first method, we use the short-time Fourier transform of the far-field radially integrated HHG spectra and distinguish between intra- and interband HHG emission according to each respective mechanism's time-frequency characteristics. Interband emission exhibits the well-known attochirp which reflects the band structure of the system, whereas intraband emission occurs at the peak of the driving electric field for all emitted frequencies.

The second method relies on the resolution of HHG emission based on lattice site separation. To demonstrate how this is accomplished, we first describe HHG using the adiabatic Houston basis and expand the time-dependent wavefunction with initial crystal momentum k_0 as follows [33]:

$$|\psi(t)\rangle = a_{m,k_0}(t)|\psi^{(H)}_{m,k_0}(t)\rangle. \tag{3.27}$$

The time-evolution of the amplitude $a_{m,k_0}(t)$ is given by

$$i\frac{\partial a_{m,k_0}}{\partial t} = \sum_{m'} \left[\epsilon_{m'}(k_0 + A(t))\delta_{m,m'} - F(t) d_{m,m'}(k_0 + A(t))\right] a_{m',k_0}(t), \tag{3.28}$$

where $\epsilon_{m'}(k)$ and $d_{m,m'}(k)$ are the energy of band m and transition dipole moment between bands m and m' at crystal momentum k, and $F(t)$ and $A(t)$ are the external time-dependent electric field and vector potential at time t. Assuming negligible depletion of the ground state, the intra- and interband current calculated from this model are given as follows:

$$j^{(ra)}(t) = -\sum_m |a_{m,k_0}(t)|^2 \langle\psi_{m,k_0+A(t)}|\hat{p}|\psi_{m,k_0+A(t)}\rangle, \tag{3.29}$$

$$j^{(er)}(t) = -\sum_{m\neq m'} \left[a^*_{m,k_0}(t)a_{m',k_0}(t)\langle\psi_{m,k_0+A(t)}|\hat{p}|\psi_{m',k_0+A(t)}\rangle + c.c.\right]. \tag{3.30}$$

We transform our representation of the system into the Wannier basis using Eq. (3.13), resulting in the following expression for the intraband emission spectrum [34]:

$$\tilde{j}^{(ra)}_{\Delta_R}(\Omega) = \sum_m \int_{-\infty}^{\infty} dt\, |a_{m,k_0}(t)|^2\, e^{-i(k_0+A(t))\Delta_R} p^{\Delta_R}_{m,m} e^{i\Omega t}. \tag{3.31}$$

We solve the integral over t in Eq. (3.31) using the saddle-point approximation, yielding the following saddle-point condition [34]:

$$0 = \Omega - F(t_s)\Delta_R \Longrightarrow N \propto \Delta_R, \tag{3.32}$$

which shows there is a minimum separation $\Delta_{R,min}$ for intraband emission at photon energy Ω to occur:

$$\Delta_{R,min} \geq \frac{\Omega}{F_0}. \tag{3.33}$$

Thus, the intraband HHG emission above the minimum band gap will be associated with lattice site separations that satisfy Eq. (3.32). In contrast, the interband emission in Eq. (3.29) depends on the interband momentum matrix element, which rapidly decays with lattice site separation. Consequently, the interband HHG emission will be dominated by coherences between relatively close lattice sites.

Therefore, we conclude that the intra- and interband HHG are governed by distinct real-space dynamics, and that resolving the HHG emission above the minimum band gap according to lattice site separation provides a means for distinguishing between the two mechanisms

3.3.3 Macroscopic Simulation Results

We now use this insight to analyse the combination of microscopic and macroscopic simulations. To make the macroscopic simulations feasible, we use a simple one-dimensional microscopic model of a crystal, with the periodic potential adjusted to obtain the desired band gap.

We simulate HHG emission across a radially symmetric Gaussian beam-front with a beam-waist of 100 µm, peak electric field amplitude 0.014 a.u., and a pulse duration of eight optical cycles for 1200 nm driving field wavelength. Fig. 13 depicts the lattice site separation-resolved radially integrated HHG spectra, after propagating the near-field signal from a 2D sample by a distance of 1 m into the far field, assuming 10 fs dephasing time. The dashed vertical red lines denote the harmonic order corresponding to the minimum band gap. The dashed white line depicts the minimum lattice site separation for the intraband emission at a given harmonic order, calculated using Eq. (3.32). The changing interplay of the two mechanisms across the harmonic spectrum is clear, with the highest harmonics dominated by the inter-band mechanism and the lower to mid-range harmonics demonstrating comparable contributions from both.

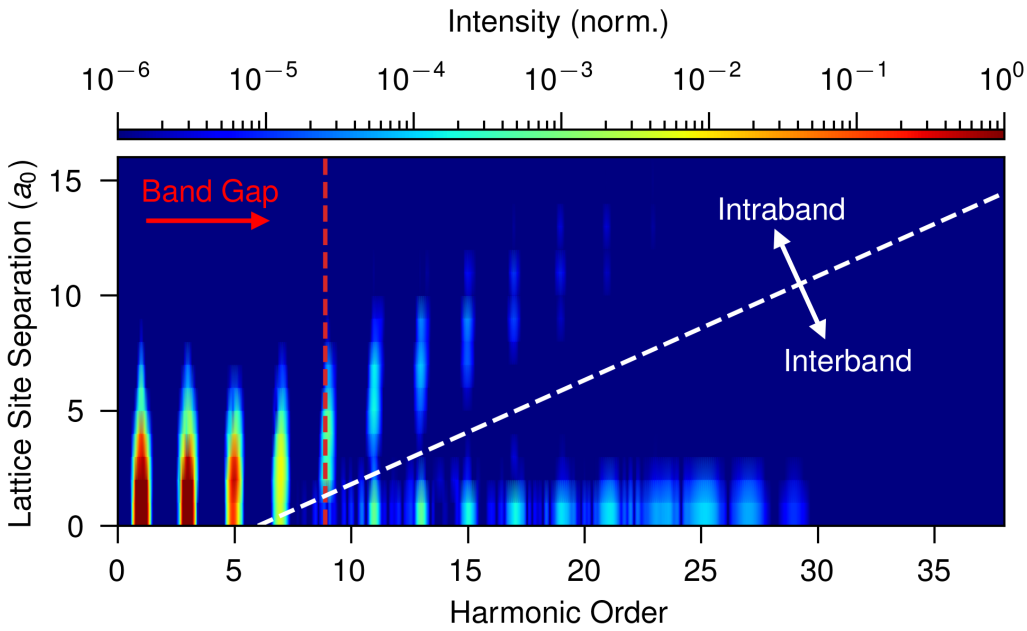

Figure 13: Lattice site separation-resolved far-field HHG spectra calculated with driving laser wavelengths of 1200nm, driving field beam waist of σ = 100 µm and a driving electric field amplitude of 0.014 a.u.. A far-field propagation distance of 1 m is considered. The dashed white lines denote the minimum lattice site separation for HHG emission at a given harmonic order calculated using Eq. (3.32) and the vertical dashed red lines denote the harmonic order of the minimum band gap.

We are now able to study the relative importance of the two HHG mechanisms on the driving field wavelength. Figure 14 presents the HHG spectrum as a function of lattice site separation for wavelengths of 0.6 µm, 0.8 µm, 1.0 µm, and 1.2 µm.

For longer driving field wavelength (c and d), the intra- and interband components of the spectrum are clearly separated above the minimum band gap, where the emission is clearly dominated by the near-zero lattice separation emission and is predominantly interband. For shorter wavelengths as well (panels (a) and (b)) the situation is different. Reducing the wavelength increases the strength of the intraband emission, and the separation between intra- and interband emission in the total HHG spectrum is less clear. At a shorter wavelength, 0.6 µm (a), the intraband emission is the dominant HHG mechanism.

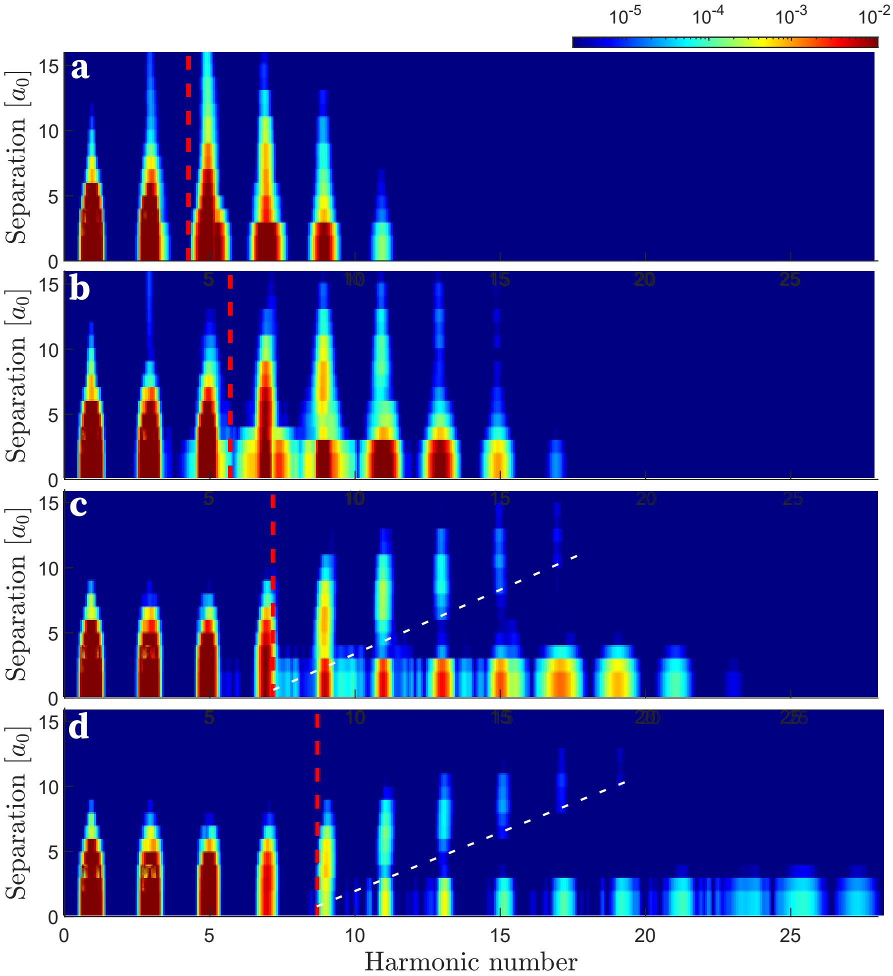

Figure 14: (a-d) Lattice site separation-resolved far-field HHG spectra, calculated with driving laser wavelengths of 0.6 µm, 0.8 µm, 1.0 µm, and 1.2 µm, respectively. All spectra are calculated with a driving field beam waist of $\sigma = 100$ µm, a far-field propagation distance of 1 m, a peak driving electric field amplitude of 0.014 a.u., and are normalized for clarity. The dashed white lines denote the minimum lattice site separation for HHG emission at a given harmonic order calculated using Eq. (3.32) and the vertical dashed red lines denote the harmonic order of the minimum band gap.

This conclusion is supported by the time-frequency Gabor analysis of the emission shown in Fig. 15, for the same driving laser wavelengths of (a) 0.6 µm, (b) 0.8 µm, (c) 1.0 µm, and (d) 1.2 µm. All Gabor transforms are normalized according to their respective maxima for photon energies above the minimum band gap. For the driving laser wavelength of 0.6 µm in (a), where the HHG emission is dominated by the intraband mechanism, no attochirp is observed, as expected.

As we go to longer wavelengths, the attochirp becomes more pronounced. The

above-band-gap emission calculated with a 1.2 μm driving laser wavelength shown in (d) exhibits a clear attochirp for nearly all photon energies above the minimum band gap, showing the dominant role of the interband emission. The emission between harmonic orders H14 and H21, those used for analysis in our experiment, clearly lie within the energy region which exhibits a clear attochirp.

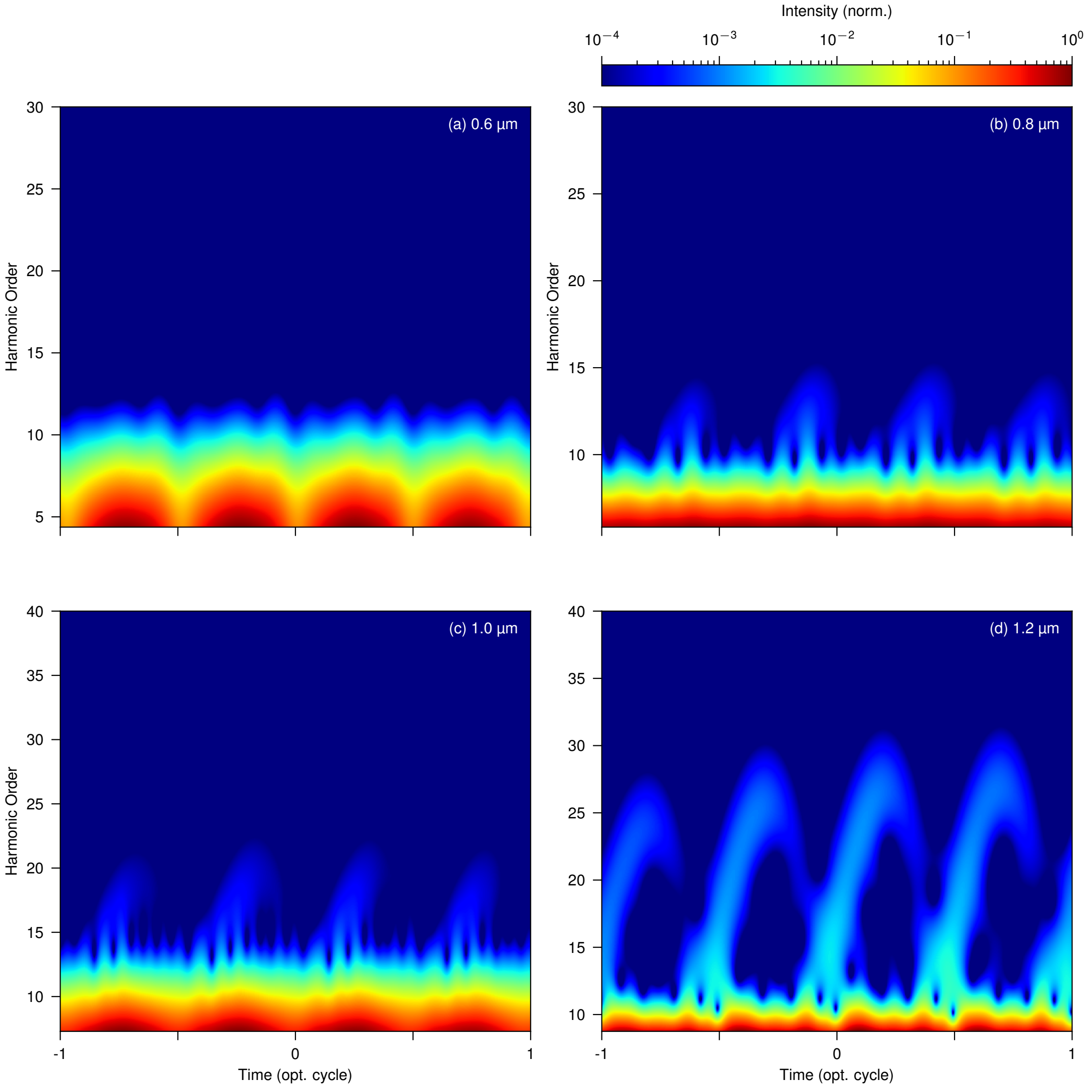

Figure 15: The short-time Fourier transforms of the radially integrated far-field HHG spectra shown in Fig. 14 (a-d) calculated with driving laser wavelengths of (a) 0.6 μm, (b) 0.8 μm, (c) 1.0 μm, and (d) 1.2 μm showing the above-band-gap HHG emission. All spectra are normalized to their peak above-band-gap emission.

Our results clearly show that the dominant mechanism of HHG emission shifts

from intra- to interband as the driving laser wavelength is increased and that interband emission is the dominant HHG mechanism for the experimental conditions used in this work.

At the same time, our results show that experiments performed with wavelengths below 800 nm will have very significant or dominant contribution from the intra-band mechanism, for the large band-gap materials such as quartz.

3.4 Physical interpretation of the interplay between inter-band and intra-band mechanisms under different conditions

Intraband currents are initiated by the injection of the charge across the band gap at t_i. These currents are proportional to the number of injected charge carriers, the density $n_c(t_i)$, and their velocity $v(t, t_i)$ dictated by the band dispersion, $j(t, t_i) = n_c(t_i)v(t, t_i)$. In contrast to the intraband currents, the interband contribution is given by the coherence created between the valence and the conduction bands upon electron injection. A simplified expression for the induced polarization is: $P(k; t) = a_c(k, t, t_i)a_v(k, t, t_i)e^{-i\int_{t_i}^{t} \epsilon(k(\tau))d\tau}$. This expression assumes a fully coherent quantum evolution between injection at t_i and observation at t. Here $a_c(k, t)$, $a_v(k, t)$ are the density *amplitudes*, with $n_c = |a_c|^2$, $n_v = |a_v|^2$.

The main difference between the two processes is simple: in the intraband case the process is dominated by the charge density while the interband process is dominated by coherence. For small injection probabilities per half-cycle, coherence scales with the **square root** of the injection probability per half-cycle, while the intraband competitors scale with the **full** probability. Therein lies the possibility to understand when one or the other dominate. In the case of low frequency driving field and long laser pulses, the dominant mechanism for charge injection into the conduction band is tunneling. In this regime, and for long laser pulses, the injection via optical tunnelling per laser cycle is exponentially low. Therefore, the intraband contributions becomes negligible while the interband contribution dominates the interaction. For few-cycle laser pulses and higher frequencies, the injection rate per laser cycle is necessarily much higher, making the intraband mechanism highly competitive. Since the intraband mechanism also does not suffer from dephasing,

it can dominate the resulting far-field emission.

Let us also look at it from another perspective. Harmonic generation, like any parametric process, requires the system to return to its initial state. This applies to both inter-band and intraband contributions. This condition is very clear in the case of interband process (figure 16a). The electron in the conduction band, which tunneled across the band gap with an amplitude a_{T}, returns with higher energy and recombines across the gap with the hole ($\langle\Psi_v(k,t)|\hat{d}|\Psi_c(k,t)\rangle$ + c.c.). This process is composed of only one tunneling step and scales with a_{T}. However, the intraband trajectory also must return to the initial state (figure 16b). We can express this condition as a two step process, where the two steps are time-reversed to each other ($\langle\Psi_c(k,t)|\hat{d}|\Psi_c(k,t)\rangle$ + c.c.). The electron tunnels across the band-gap, moves inside the conduction band, and emits harmonic light during this motion. This step, described by $|\Psi_c(k,t)\rangle$, scales with the tunneling amplitude (a_{T}). Then, the electron *traces its route backwards in time, including the tunneling step*, which is described by $\langle\Psi_c(k,t)|$ and scales with the complex conjugated amplitude a_{T}^*. In this case the initial and the final states are both associated with the conduction band and therefore they contain the tunnelling amplitude on the both sides: the bra- and the ket- sides (The bra-side is time-reversed to the ket). The result scales with tunnelling probability, $a_{\mathrm{T}}^* a_{\mathrm{T}} = |a_{\mathrm{T}}|^2$.

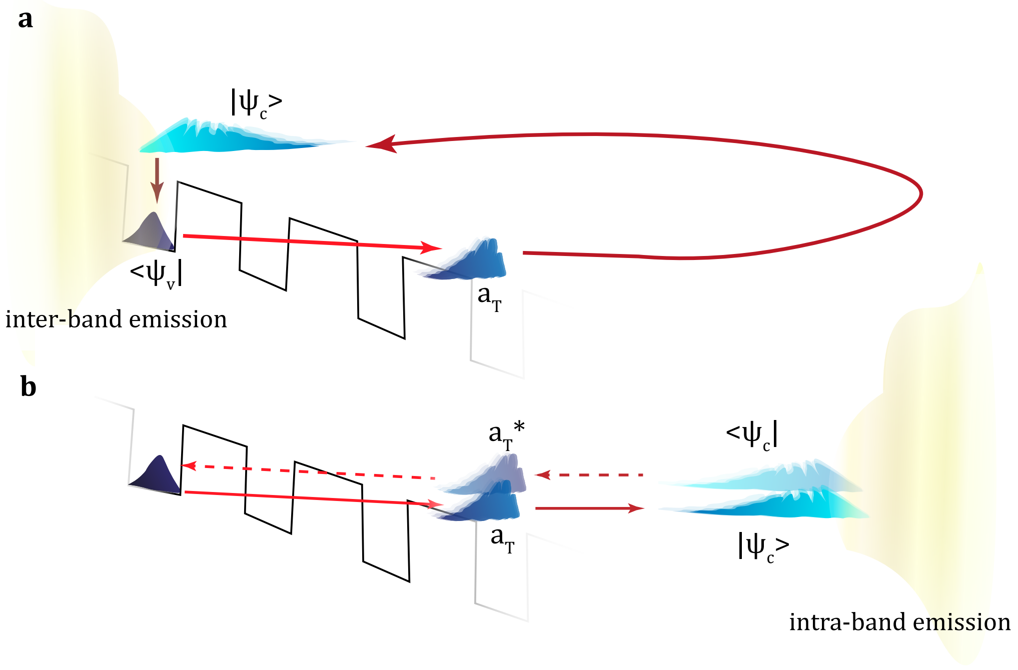

Figure 16: **Illustration of the interband and intraband mechanisms.** (a), In the interband picture, the electron tunnels with an amplitude a_T, evolves $(|\Psi_c(k,t)\rangle)$ and returns to the origin $(\langle\Psi_v(k,t)|)$, emitting a photon. (b) In the intraband picture, the electron tunnels with amplitude a_T, evolves $(|\Psi_c(k,t)\rangle)$ and returns to the initial state according to the time reversal process $(\langle\Psi_c(k,t)|)$, acquiring additional tunneling amplitude a^*_{T} .

Thus, as soon as we reduce the frequency of the driving field to bring the interaction into tunnelling or the non-adiabatic tunnelling regime, the interband contribution should dominate the intraband one for high harmonics: the second tunnelling step, back from the conduction to the valence band, is not needed for the interband emission. This explains why the interband contribution usually dominates in the low-frequency driving regime campared to high-frequency driving field [13].

To conclude, the inter-band contribution naturally dominates in the low-frequency regime of optical tunnelling, whether adiabatic or non-adiabatic, because it scales with the tunnelling amplitude per half-cycle and not the tunnelling probability per half-cycle. Detrimental effects of ionization on the macroscopic high harmonic generation strongly limit tunnelling probability per half-cycle in the multi-cycle, long-pulse regime. The intraband contribution can, however, dominate for high harmonics in the nearly single-cycle pulse regime. Moreover, the combination

of high-frequency driving field and single cycle regime can lower the tunnelling penalty and lead to the contribution of the intraband to be further enhanced.

3.5 The physics of real and virtual excitations in the strong field regime and their relative importance in HHG

In our study, described in chapter 3.2, we focus on the harmonics emitted from the first conduction band. In this chapter we discuss the contribution of higher conduction bands in our measurements. The role of higher conduction bands crucially depends on the nonlinearity of the light matter interaction. In perturbative nonlinear optics, nonlinear susceptibilities responsible for harmonic generation always include summation over all possible intermediate virtual states – infinitely many of them, in fact. Thus, why can one focus on a particular conduction band? The same question applies to HHG in atoms (molecules, etc) – why do we describe a particular high harmonic emission, $N\omega_0$, in strong laser fields, via one-photon recombination from a continuum state with the energy E given by the energy conservation law, $N\omega_0 = E + I_p$ (I_p being the ionization potential)? Why do we not include the integral over all other bound and continuum states which, according to perturbative nonlinear optics, should also contribute?

The rigorous answer is by now well-known: in the strong-field regime, where the electron action is very large, the corresponding integrals over all quantum paths (all states) are dominated by the semi-classical paths which correspond to real excitations, selecting the state with energy $E = N\omega_0 - I_p$ out of all possible recombination states. This selection becomes possible thanks to the strong laser field and the very large action accumulated by the electron between ionization and recombination. The formal mathematical analysis of the arising Green's function integrals in the time-domain is taken using the saddle point method, leading to the concept of quantum trajectories and selecting a well-defined momentum state for the generation of a particular harmonic. This answer applies to both atoms and solids, to both inter-band and intra-band harmonics.

One should always remember the physics described by the virtual excitatations and by the contributions of far-away virtual states: they describe laser-induced polarization of the active band (or state), its distortion by the instantaneous laser

field. Thus, other bands are not irrelevant: they do contribute by describing field-induced modifications of the conduction band of interest. Of course, this modification is fully included in all our simulations and it does not in any way affect our concept of the interband Berry phase, which is accumulated during the evolution in the laser-polarized conduction and valence bands. Such band polarization might somewhat shift the emission point for a particular harmonic, due to the distortion of the band, but in no way would it affect our general derivation of the interband Berry phase.

To summarize this point: we are in the strong field regime, where we have the luxury of dealing with real excitations, which dominate virtual excitations. In contrast, virtual excitations dominate in the weak field regime, where no real excitations occur. The demarcation line between the two regimes is clearly visible in the harmonic spectra and is associated with the long harmonic plateau, which is clearly the case here.

This demarcation applies to both intra-band and inter-band harmonics: once real injection of electrons in the conduction band occurs, all harmonics are dominated by these real excitations and not by virtual ones. The most stringent test on the relative role of virtual and real excitations is done when studying the lower order harmonics, where the Brunel type lower-order nonlinearities associated with real excitations are directly competing with the Kerr-type nonlinearities of the same order, associated with virtual excitations. This transition between the two regimes has been specifically studied in solids by P Jürgens, et al, [35]. Their conclusion was summarized above.

This is why in our study we focus on the specific range of harmonics emitted from the first conduction band (harmonics H11-H17), even though our two color spectrum spans from H11 to H31: we wanted to make sure that the underlying dynamics is relatively straightforward and does not include significant real excitations to higher conduction bands. As for the virtual transitions, they are responsible for the distortion of the bands but do not change the concept of the interband Berry phase in any way. As stated above, all of this complexity is fully included in the numerical analysis.

It is worthwhile to note that propagation to higher conduction bands in the strong field regime would involve additional tunneling steps. Crucially, the concept

of the interband Berry phase introduced here will remain fully valid throughout – only the pathways would become more complex, spanning multiple bands. While these processes encode rich and complex dynamics, they are beyond the scope of this paper (see our recent publication [26] for pertinent analysis).

4 Semi classical trajectory analysis

In order to evaluate the role of Berry curvature in the electron-hole dynamics, we perform a semi-classical 2D trajectory simulation in real space. We calculate the electron-hole trajectories that evolve within a single cycle of the driving electric field, along the Γ-K or Γ-M directions. The driving field was set at a wavelength of $1.3\,[\mu m]$ and an intensity of $0.007\,[a.u.]$. Our calculations were performed using driving field with a wavelength of $1.2\,[\mu m]$ due to technical reasons only and we expect them to be very similar to $1.3\,[\mu m]$ driving field case. The band structure and Berry curvature are calculated via density functional theory (DFT) calculations (see fig. 18) with an inner valence band chosen to avoid band crossings that affect the numerical error. The dynamics of the electron-hole pair is governed by the semi-classical equations of motion for solids (atomic units applied) [8]:

$$\left\langle \dot{\boldsymbol{k}}\left(t\right)\right\rangle = -\boldsymbol{F}\left(t\right) \tag{4.1}$$

$$\left\langle \boldsymbol{v}\left(t\right)\right\rangle = \nabla_{\boldsymbol{k}}\varepsilon_{cv}\left(\boldsymbol{k}\right)\big|_{\boldsymbol{k}=\langle\boldsymbol{k}(t)\rangle} + \left\langle \dot{\boldsymbol{k}}\left(t\right)\right\rangle \times \boldsymbol{\Omega}_{cv}\left(\boldsymbol{k}\left(t\right)\right) \tag{4.2}$$

where ε_{cv} is the bandgap between conduction and valence bands, while $\boldsymbol{\Omega}_{cv}$ is the Berry curvature contribution of the electron (conduction) band and the hole (valence) band defined as:

$$\varepsilon_{cv}\left(\boldsymbol{k}\right) \equiv \varepsilon_{c}\left(\boldsymbol{k}\right) - \varepsilon_{v}\left(\boldsymbol{k}\right) \tag{4.3}$$

$$\boldsymbol{\Omega}_{cv}\left(\boldsymbol{k}\right) \equiv \boldsymbol{\Omega}_{c}\left(\boldsymbol{k}\right) - \boldsymbol{\Omega}_{v}\left(\boldsymbol{k}\right) \tag{4.4}$$

$\langle\boldsymbol{v}(t)\rangle$ in eq. (4.2) is the expectation value of the relative velocity operator between the electron and the hole. The first term on the right hand side of eq. (4.2) represents the relative group velocity calculated at specific momentum $\boldsymbol{k}$ in the

band, while the second term is the anomalous velocity, governed by the Berry curvature.

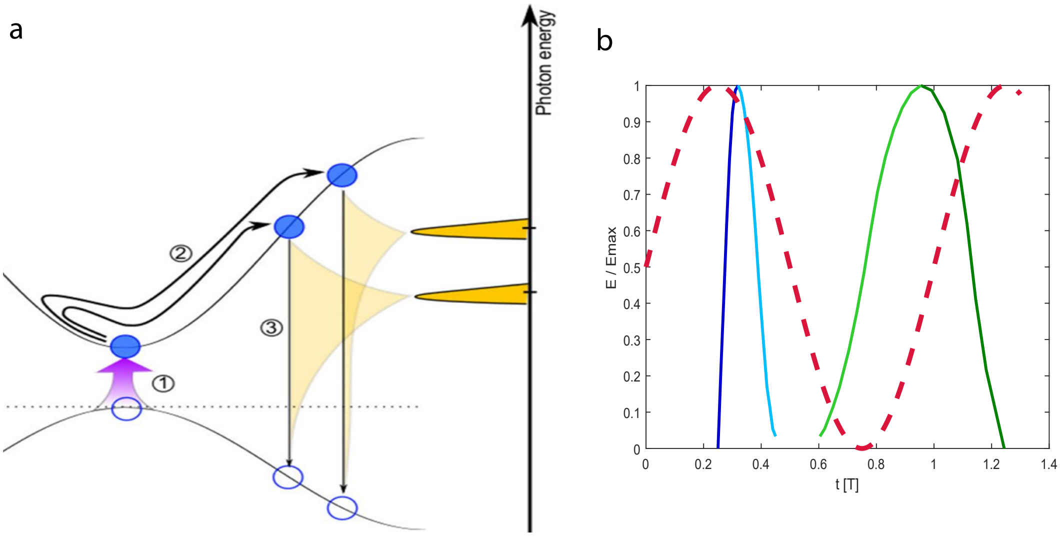

Figure 17: **Time-Energy mapping:** **(a)** Schematic description of interband mechanism: (1) an electron is tunnel ionized at time t', leaving a hole in the valence band. Then, (2) both propagate, until (3) overlapping in real space and recombining at time t. The emitted photon energy is equal to the band gap energy at the recombination moment (picture taken from [36]). Different trajectories lead to the measured broad emission spectrum. **(b)** The ionization times (blue) and recombination times (green) of the different trajectories that lead to their corresponding emitted photon (y axis), in terms of the driving field period (dashed red). The light and dark colors distinguish between two families of trajectories, short and long [37]. In our analysis we consider the short trajectories only, characterised by later ionization times and shorter recombination times.

Our analysis is based on the interband model [38]. Under the influence of the strong laser field an electron tunnels from the valence band into the conduction band, initiating an electron-hole wavepacket. Tunneling evolves at the Γ point in the Brillouin zone, where the crystal momentum is zero. As the electric field evolves, the electron-hole pair travels in the Brillouin zone, acquiring crystal momentum according to eq. (4.1). The crystal momentum is then translated into real-space displacement, dictated by the band's dispersion and the Berry curvature (eq. (4.2)). When the electron and hole wave-packets overlap in real space, recombination occurs, emitting a photon with energy equal to the energy difference between the conduction and the valence bands, at the instant of recombination:

$$\Delta r(t', t) = \int_{t'}^{t} v(\tau) d\tau = 0 \tag{4.5}$$

$$\varepsilon_{cv}(k(t)) = \hbar\omega \tag{4.6}$$

Where (t',t) are the tunneling and recombination times, $k(t)$ is the crystal momentum at the recombination moment and $\hbar\omega$ is the photon energy. Note that the recombination is induced when the electron and hole overlap in real space within the characteristic electron's wave-packet width. We evaluate the wave-packet's width by following experimentally the decay of the harmonics signal with the fundamental field's ellipticity. This width is defined by ellipticity at which the harmonics signal approaches FWHM level. We consider a set of possible tunneling times (t'), leading to different k space/real space trajectories and recombination events (t, ω). This provides the mapping between the measured photon emission, resolved in the HHG spectrum, to the underlying k space dynamics (figure 17a)[12]. In figure 17b we present this calculated mapping, according to our semiclassical analysis described above, considering a linear field. This figure describes, for each photon energy, the ionization time and recombination time.

To fully evaluate the effect of the Berry curvature on the trajectories, we consider three different cases:

1. Single-color linearly-polarized light
2. Single-color elliptically-polarized light
3. Two-color cross-polarized light

In the following sections we provide a detailed analysis of these cases and extract their response to the Berry curvature.

4.1 Single-color linearly-polarized light

We perform the simulations for a single-color, linearly-polarized driving electric field ($A^y = A_0^y \sin(\omega_0 t)$), calculating the electron-hole dynamics in both half cycles. Figure 19 illustrates the real space trajectories evolving after the ionization step for two consecutive half cycles. Recombination occurs at the second time when the trajectory intersect with $y = 0$ (the relative distance between the electron and the hole vanishes). We focus on two important cases: i) the Γ-K direction, where the Berry curvature vanishes; and ii) the Γ-M direction, where the Berry curvature is maximal. For the Γ-K direction, we observe an analogous behavior to an inversion-symmetric material: no transverse displacement (x direction, blue line) occurs with

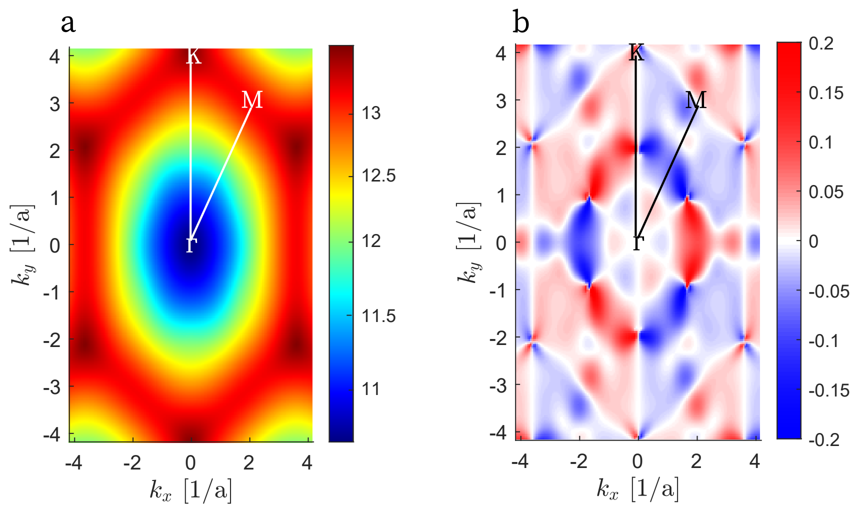

Figure 18: DFT calculation of **(a)** the energy gap ε_{cv} (units of eV) and **(b)** Berry curvature difference Ω_{cv} (units of $\frac{1}{a^2}$, a being the lattice constant) over the first Brillouin zone, for the lowest conduction band and an inner valance band.

respect to the field's polarization (y direction) (Fig. 19 top panel). In contrast, for the Γ-M direction, we observe a significant transverse displacement along the x direction. The directionality of this displacement is preserved between the two half cycles, since both the electric field and the Berry curvature invert their sign (Fig. 19 bottom panel).

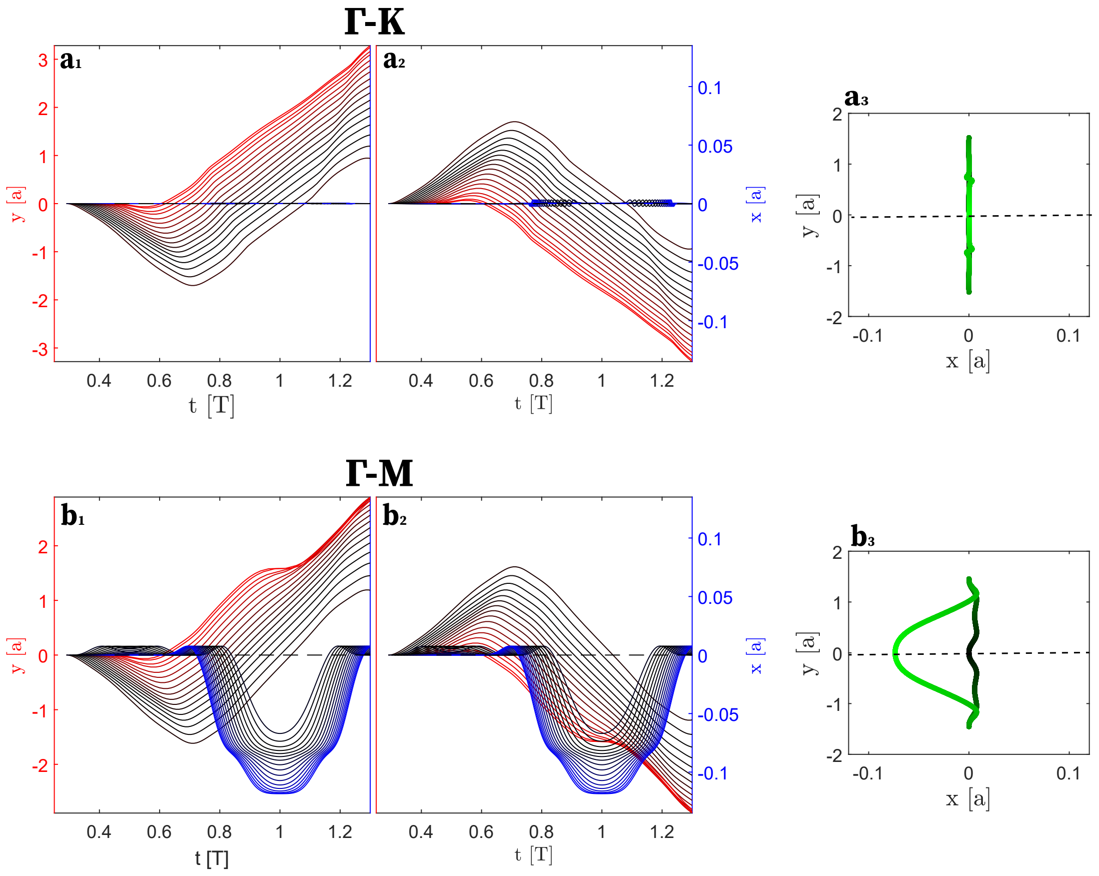

Figure 19: Electron-hole trajectories, driven by a single-color linearly-polarized light, along the Γ-K (upper panel, **a1:a2**) and Γ-M (lower panel, **b1:b2**) directions for each half-cycle of the field. a1 (a2) and b1 (b2) represent the trajectories along positive (negative) subcycle. The relative displacement between electron and hole is shown in units of lattice constant. The left axis (red) is the y displacement (along the fundamental field's polarization), while the right axis (blue) is the x displacement. The brightness of the different plots encodes the mapping to recombination energy: light (dark) colored trajectory will be map to low (high) recombination energy as well as to low (high) emitted photon energy. The trajectories are drawn beyond the recombination time to provide a schematic visualization of their response. **a3, b3** x-y cross-sectional view of a one chosen trajectory for Γ-K and Γ-M orientations, during the evolution along the two subcycles. The varying color encodes time from ionization (black) to recombination (green).

4.2 Single-color elliptically-polarized light

In the next stage, we simulate the trajectories driven by a single-color elliptically-polarized light $(\epsilon A_0^y \cos(\omega_0 t), A_0^y \sin(\omega_0 t))$. In the absence of Berry curvature (i.e., along the Γ-K direction), the displacement of the electron-hole pair in the transverse direction (x) changes sign between two consecutive half cycles. In the presence of Berry curvature (i.e., along the Γ-M direction), for small epsilons, the

trajectories of both half cycles stay similar, with only the accumulated phase being different due to the flip in the Berry curvature sign (see fig. 20).

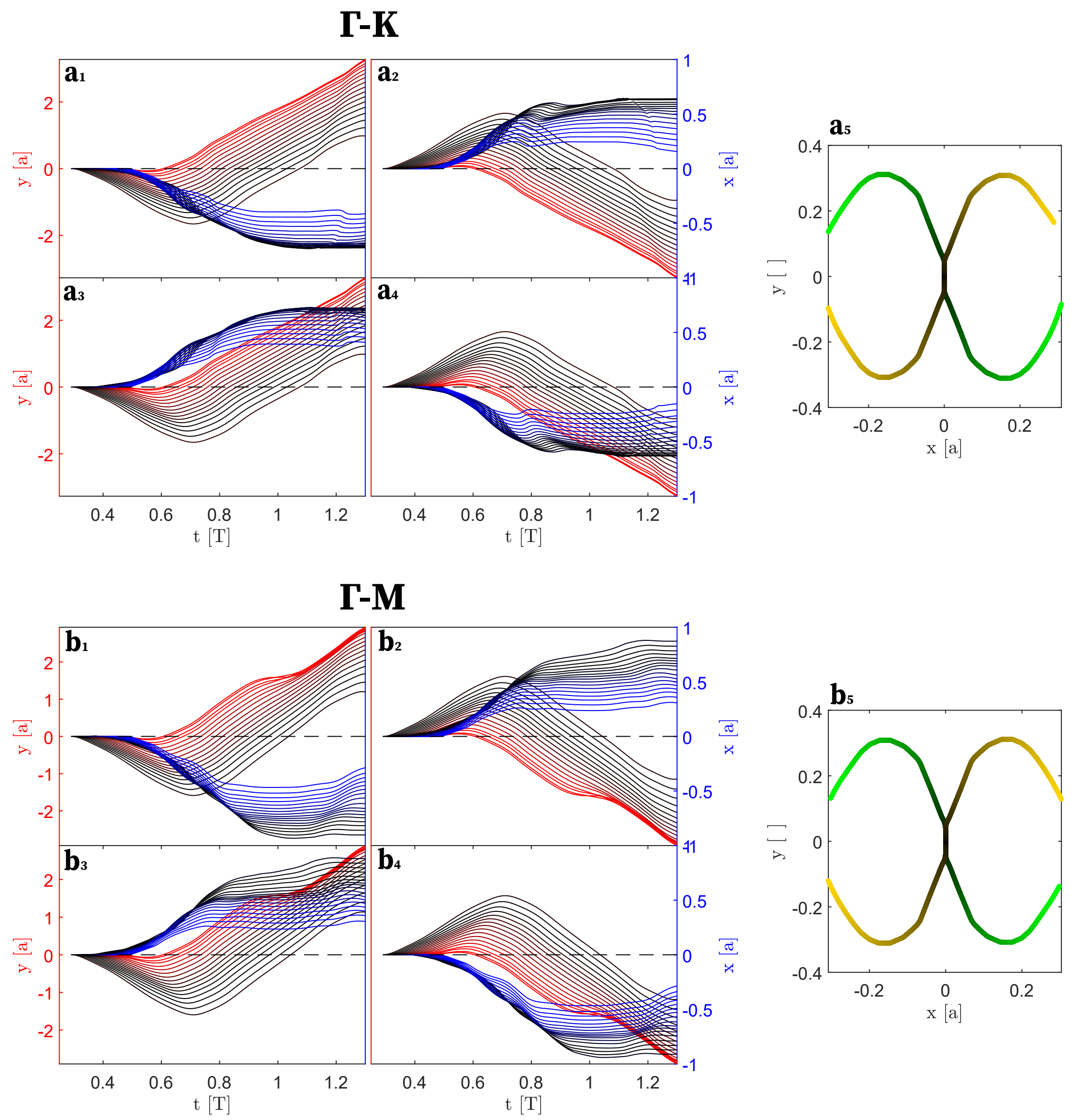

Figure 20: Electron-hole trajectories, driven by an elliptically polarized single-color field, along the Γ-K (upper plots) and Γ-M (lower plots) directions for $\epsilon = 0.1$ (**a1,a3, b1,b3**) and $\epsilon = -0.1$ (**a2,a4, b2,b4**) with each subplot representing a half-cycle of the field. a1,b1 (a2,b2) and a3,b3 (a4,b4) represent the trajectories during a positive (negative) subcycle. The relative displacement between electron and hole in units of lattice constant are shown: The left axis (red) is the y displacement while the right axis (blue) is the x displacement. The brightness of the different plots encodes the mapping to recombination energy. Light (dark) colored trajectories are mapped to low (high) recombination energies as well as to low (high) emitted photon energies. The trajectories are drawn beyond the recombination time to provide a schematic visualization of their response. **a5, b5** x-y plot of a single chosen trajectory for $\epsilon = \pm 0.1$ (green) and $\epsilon = -\pm 0.1$ (gold), during both subcycles. The brightness encodes the time evolution from ionization (dark) to recombination (light).

4.3 Two-color cross-polarized light

Finally, we simulate the trajectories under the influence of a cross-polarized two-color light, $(\epsilon A_0^y \sin(2\omega_0 t + \varphi), A_0^y \sin(\omega_0 t),)$, where φ is the two fields subcycle delay. The second harmonic (SH) field amplitude is 5% of the fundamental field's amplitude. In this case, the transverse displacement is dictated by the presence of the SH field, which exerts a transverse force (i.e., in the x direction) as well as the Berry curvature. This force can either enhance or reduce the transversal displacement, induced by the anomalous velocity, *for both subcycles.* For each crystal orientation, Γ-K and Γ-M, we describe the trajectories, for two color delays of -0.5π and 0.5π (see fig. 21). For the Γ-K direction (no Berry curvature), the transverse displacement amplitude (x-direction, blue line) is invariant to an integer multiple of a π phase shift (see fig. 21, upper panel). Along this direction the Berry curvature vanishes, therefore no anomalous velocity arises. Scanning the two color delay will lead to a periodic modulation of the recombination signal, and therefore of the HHG signal, at twice the SH frequency. Conversely, for the Γ-M direction (maximal Berry curvature), the transverse displacement is suppressed for the -0.5π phase shift, but enhanced for the 0.5π phase shift (see fig. 21, lower panel). This observation occurs symmetrically for both half cycles, since the two-color field features the same symmetry between half-cycles, as it is for the anomalous velocity. Such response breaks the symmetry between $\varphi = -0.5\pi$ and $\varphi = 0.5\pi$, reducing the periodicity of the two color delay to be a full period of the SH field.

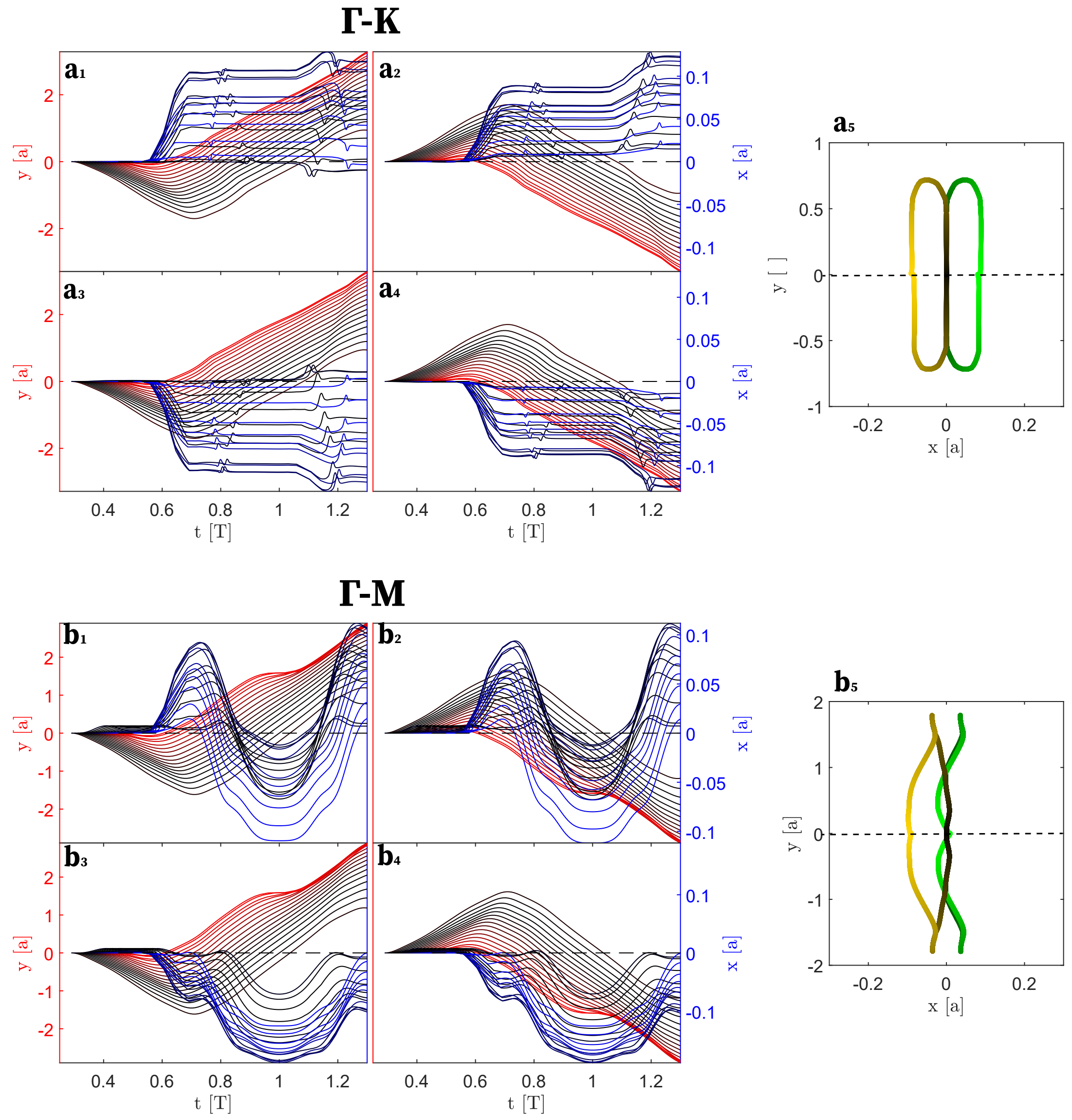

Figure 21: Electron-hole trajectories, driven by a two-color orthogonally-polarized field, along the Γ-K (upper panel) and Γ-M (lower panel) directions for $-\pi/2$ (**a1-a2, b1-b2**) and $\pi/2$ (**a3-a4, b3-b4**) two color delay. Each subplot represents a half-cycle of the field. a1,b1 (a2,b2) and a3,b3 (a4,b4) represent the trajectories during a positive (negative) subcycle. The relative displacement between electron and hole is shown in units of lattice constant. The left axis (red) is the y displacement, while the right axis (blue) is the x displacement. The brightness of the different plots encodes the mapping to recombination energy. Light (dark) colored trajectories are mapped to low (high) recombination energies as well as to low (high) emitted photon energies. The trajectories are drawn beyond the recombination time to provide a schematic visualization of their response. **a5, b5** x-y plot of a single chosen trajectory for $-\pi/2$ (green) and $\pi/2$ (gold) two color delays, during both subcycles. The brightness encodes the time evolution from ionization (dark) to recombination (light) time.

5 HHG Spectroscopy in broken inversion crystals-Quantum analysis

We start with a general description of HHG spectroscopy, resolved in a non-inversion symmetric crystal, adapted from ref [39]. We focus on the interband (recombination) picture, considering a two-band system. As was described in chapter 3, the interband mechanism is the dominant mechanism in our experimental conditions [23, 39]. While the following description considers a two-band system, we note that it can be easily extended to a multiband system[23]. Next, we present the semi-classical limit [37] of this quantum study, coinciding with the semi-classical analysis of Bloch electrons (chapter 4). Finally, we present a broad discussion concerning the interband Berry phase, as a manifestation of discrete geometrical phase. This includes its mathematical formalism and the relation to HHG spectroscopy.

5.1 Interband emission

A quantum description [37] of the inter-band mechanism is obtained by solving the Semiconductor Bloch Equations (SBE) using so-called Keldysh approach, introduced by Vampa et al. [38]. According to this approach, the interbnad current is dictated by a coherent sum over all quantum paths, weighted by their dipole coupling and the semi-classical action:

$$\mathbf{J}_{inter}(\omega) = i\int_{BZ} d^3K \int_{-\infty}^{t} dt' \int_{-\infty}^{\infty} dt \mathbf{F}(t') \cdot \mathbf{d}(\mathbf{K}+\mathbf{A}(t'))\mathbf{d}^*(\mathbf{K}+\mathbf{A}(t))e^{-iS[\mathbf{K},t',t]+i\omega t} + c.c \tag{5.1}$$

Where $S[\mathbf{k}, t', t]$ is the semi-classical action which in a broken inversion system contains the band dispersion as well as the Berry connection:

$$S[\mathbf{K}, t', t] \equiv \int_{t'}^{t} [\varepsilon_g(\mathbf{K}+\mathbf{A}(\tau)) + \mathbf{F}(\tau) \cdot \boldsymbol{\mathcal{A}}_g(\mathbf{K}+\mathbf{A}(\tau))]d\tau \tag{5.2}$$

Here $\mathbf{d}(\mathbf{k}) = i\langle u_{v,\mathbf{k}}(x)| \nabla_{\mathbf{k}} |u_{c,\mathbf{k}}(x)\rangle$ is the dipole coupling between the valence and the conduction bands, $u_{v/c,\mathbf{k}}(x)$ is the periodic part of the Bloch functions ($\psi_{n,k} =$

$e^{ik\cdot r}u_{n,k}(r)$) and $\varepsilon_g = \varepsilon_c - \varepsilon_v$ is the two bands energy difference. $\boldsymbol{\mathcal{A}}_g = \boldsymbol{\mathcal{A}}_c - \boldsymbol{\mathcal{A}}_v$ is their relative Berry connection, defined as $\boldsymbol{\mathcal{A}}_{\mathbf{m}}(\mathbf{k}) = i\langle u_{m,\mathbf{k}}(x)|\nabla_{\mathbf{k}}|u_{m,\mathbf{k}}(x)\rangle$, in which $m = v, c$. $\mathbf{F}(t)$ and $\mathbf{A}(t)$ are the laser field and its vector potential and $\mathbf{K(t)} = \mathbf{k} - \mathbf{A}(t)$ is the canonical crystal quasi-momentum.

The system symmetries dictate the complex properties of the dipole coupling. Time reversal symmetry leads to $\mathbf{d}(\mathbf{-k}) = \mathbf{d}^*(\mathbf{k})$ and inversion symmetry to $\mathbf{d}(\mathbf{-k}) = -\mathbf{d}(\mathbf{k})$. Therefore, the dipole coupling induced in a crystal with inversion symmetry is purely imaginary. However, in a broken inversion crystal, the dipole coupling is a complex quantity, composed by real and imaginary components. Adding the dipole phases to the action, defining the *generalized action*, provides a gauge invariant quantity, as presented in the main text (Equation 1):

$$\begin{aligned} J^{(i)}_{inter}(\omega) &= i\int_{BZ} d^3K \int_{-\infty}^{t} dt' \int_{-\infty}^{\infty} dt F^{(j)}(t')\cdot|d^{(j)}(\mathbf{K}+\mathbf{A}(t'))||d^{*(i)}(\mathbf{K}+\mathbf{A}(t))|e^{-iS^{(i)}[\mathbf{K},t',t]+i\omega t} + c.c \\ S^{(i)}[\mathbf{K},t',t] &\equiv \int_{t'}^{t} [\varepsilon_g(\mathbf{K}+\mathbf{A}(\tau)) + \mathbf{F}(\tau)\cdot\boldsymbol{\mathcal{A}}_g(\mathbf{K}+\mathbf{A}(\tau))]d\tau + \phi_d^{(j)}(\mathbf{K}+\mathbf{A}(t')) - \phi_d^{(i)}(\mathbf{K}+\mathbf{A}(t)) \end{aligned} \tag{5.3}$$

Where ϕ_d is the dipole coupling phase and $j, i = x, y$. We separate the action to its trivial component, which controls the interaction in inversion symmetric crystal and to the gauge invariant Berry phase:

$$\begin{aligned} S^{(i)}[\mathbf{K},t',t] &\equiv S_{inv} + \gamma_B^{(i)} \\ S_{inv}[\mathbf{K},t',t] &\equiv \int_{t'}^{t} \varepsilon_g(\mathbf{K}+\mathbf{A}(\tau))d\tau \\ \gamma_B^{(i)}[\mathbf{K},t',t] &\equiv \int_{t'}^{t} \mathbf{F}(\tau)\cdot\boldsymbol{\mathcal{A}}_g(\mathbf{K}+\mathbf{A}(\tau))d\tau + \phi_d^{(j)}(\mathbf{K}+\mathbf{A}(t')) - \phi_d^{(i)}(\mathbf{K}+\mathbf{A}(t)) \end{aligned} \tag{5.4}$$

Due to time reversal symmetry, S_{inv} is symmetric at positive and negative crystal momentum, $S_{inv}(-\mathbf{k}) = S_{inv}(\mathbf{k})$, while the Berry phase switches its sign, $\gamma_B(-\mathbf{k}) = -\gamma_B(\mathbf{k})$. Finally, in order to simplify the time dependence of this expres-

sion we rewrite the Berry phase as:

$$\begin{aligned}\gamma_B^{(i)}[\mathbf{K},t',t] &\equiv \tilde{\gamma_B} + \Delta\phi_d^{(i)} \\ \tilde{\gamma_B} &\equiv \int_{t'}^{t} \mathbf{F}(\tau)\cdot[\boldsymbol{\mathcal{A}}_g(\mathbf{K}+\mathbf{A}(\tau)) + \nabla_K\phi_d^{(j)}(\mathbf{K}+\mathbf{A}(\tau))]d\tau \\ \Delta\phi_d^{(i)} &\equiv \phi_d^{(j)}(\mathbf{K}+\mathbf{A}(t)) - \phi_d^{(i)}(\mathbf{K}+\mathbf{A}(t))\end{aligned} \tag{5.5}$$

Note that the Berry phase contains the difference between the recombination dipole phase of the two polarization components, which becomes zero when the dipole is parallel to the fundamental field's polarization. Therefore, it is useful to define the polarization independent Berry phase, $\tilde{\gamma_B}$, which is mutual at both polarization components.

5.2 Saddle point approximation

Equation (5.3) provides an expression for the interband current generated in the HHG process, resembling the three steps in the three-step model: (i) first, the electron is tunnel-ionized by the electric field $F^{(j)}d^{(j)}\left(\boldsymbol{K}+\boldsymbol{A}\left(t'\right)\right)$ accumulating the ionization dipole phase, (ii) then, the electron propagates inside the bands, accumulating a phase $e^{-iS\left[\boldsymbol{K},t',t\right]}$ (iii) finally, the electron recombines with its parent hole $d^{*(i)}\left(\boldsymbol{K}+\boldsymbol{A}\left(t\right)\right)$, accumulating the recombination dipole phase. The recombination leads to the emission of a photon $e^{-i\omega t}$, having an energy dictated by the associated electron-hole energy gap. All quantum paths are then coherently summed over, each one corresponding to different ionization times and ionization locations in the Brillouin zone $\int_{BZ} d^3\boldsymbol{K} \int_{-\infty}^{t} dt'$. Their interference dictates the final interband current as well as the spectrum of the emitted HHG.
An intuitive physical picture of the underlying dynamics can be achieved by taking an approximation, known as the Saddle Point Approximation (SPA). When $e^{-iS\left[\boldsymbol{K},t',t\right]}$ oscillates rapidly compared to the pre-exponential factors, then only quantum paths near the stationary points of the action S contribute to the interband current. These paths are defined by a set of parameters $\{\boldsymbol{K}_{st}, t'_{st}, t_{st}\}$ for which $\nabla S = 0$, which are derived from the following stationary equations [39, 40]:

$$\varepsilon_g\left(\boldsymbol{K}+\boldsymbol{A}\left(t'_{st}\right)\right) + \boldsymbol{F}^{(j)}\left(t'_{st}\right)\mathcal{D}^{(j)}\left(t'_{st}\right) = 0 \tag{5.6}$$

$$\Delta \boldsymbol{r} - \mathcal{D}^{(i)}\left(t_{st}\right) + \mathcal{D}^{(j)}\left(t'_{st}\right) = \mathbf{0} \tag{5.7}$$

$$\varepsilon_g\left(\boldsymbol{K} + \boldsymbol{A}\left(t_{st}\right)\right) + \boldsymbol{F}\left(t_{st}\right) \cdot \left[\mathcal{D}^{(j)}\left(t'_{st}\right) + \Delta \boldsymbol{r}\right] = \omega \tag{5.8}$$

with (j) and (i) being the components parallel and perpendicular to the ionization field respectively, and:

$$\Delta \boldsymbol{r} \equiv \int_{t'_{st}}^{t_{st}} \left[\boldsymbol{v}_c\left(\tau\right) - \boldsymbol{v}_v\left(\tau\right)\right] d\tau \tag{5.9}$$

$$\boldsymbol{v}_n\left(\tau\right) \equiv \nabla_{\boldsymbol{K}} \varepsilon_n\left(\boldsymbol{K} + \boldsymbol{A}\left(\tau\right)\right) - \boldsymbol{F}\left(\tau\right) \times \boldsymbol{\Omega}_n\left(\boldsymbol{K} + \boldsymbol{A}\left(\tau\right)\right) \tag{5.10}$$

$$\mathcal{D}^{(i)}\left(\tau\right) \equiv \boldsymbol{\mathcal{A}}_g\left(\boldsymbol{K} + \boldsymbol{A}\left(\tau\right)\right) - \nabla_{\boldsymbol{K}} \phi_d^{(i)}\left(\boldsymbol{K} + \boldsymbol{A}\left(\tau\right)\right) \tag{5.11}$$

Eq. (5.10) defines two contributions to the wavepacket velocity: the band dispersion $\nabla_{\boldsymbol{K}} \varepsilon_n$, and the anomalous velocity $\boldsymbol{F} \times \boldsymbol{\Omega}_n$ due to Berry curvature, being perpendicular to the driving field.

In the following step we neglect the dipole phase contributions $\mathcal{D}^{(j)}\left(\tau\right)$, which are commonly small [40], simplifying the stationary equations to be:

$$\varepsilon_g\left(\boldsymbol{K} + \boldsymbol{A}\left(t'_{st}\right)\right) = 0 \tag{5.12}$$

$$\Delta \boldsymbol{r} = 0 \tag{5.13}$$

$$\varepsilon_g\left(\boldsymbol{K} + \boldsymbol{A}\left(t_{st}\right)\right) = \omega \tag{5.14}$$

According the first equation the electrons are born with zero crystal momentum, at time t'_{st} for zero band gap energy. The finite band gap energy results in a complex birth time, which represents the tunnel ionization process. The second equation reflects that recombination occurs when the electron and hole wavepackets overlap in real-space. Finally, the third stationary equation represents conservation of energy: the electron-hole pair recombines and emits a photon ω with an energy equal to the energy band gap at moment of recombination t_{st}.

These results are fully compatible with the semi-classical equations of motion (4.2). The recombination condition and emitted photon energy comply with equations (5.13) and (5.14), respectively. The quantum nature of HHG is captured by equation (5.12), where the tunneling process leads to an imaginary ionization time. The coupling between the different saddle point equations leads to a complex crystal

momentum and recombination time as well as to a complex Berry phase.

5.3 Adding a parallel perturbation

We theoretically add a parallel perturbation to the fundamental electric field with twice the frequency, giving a total vector potential of:

$$\boldsymbol{A}(t) = a_0 sin(\omega t)\,\hat{\boldsymbol{x}} + \epsilon a_0 sin(2\omega(t+\tau))\,\hat{\boldsymbol{x}} \tag{5.15}$$

with $\epsilon \ll 1$ and τ being the amplitude ratio and delay of the perturbation with the fundamental field, respectively. The semi-classical action from equation (5.3) becomes, to first order in ϵ:

$$\tilde{S}^{(i)}[\boldsymbol{K}, t', t, \tau] = S^{(i)}[\boldsymbol{K}, t', t] + \epsilon\sigma^{(i)}[\boldsymbol{K}, t', t, \tau] \tag{5.16}$$

with:

$$\sigma^{(i)}[\boldsymbol{K}, t', t, \tau] = \int_{t'}^{t} \Big[\Big(\nabla_{\boldsymbol{K}}\varepsilon_g\big|_{\boldsymbol{\kappa_0}(t'')} + \boldsymbol{F}_0(t'')\cdot\nabla_{\boldsymbol{K}}\boldsymbol{\mathcal{A}}\big|_{\boldsymbol{\kappa_0}(t'')}\Big) a_0\left(sin(2\omega(t''+\tau)) - sin(2\omega(t+\tau))\right) \tag{5.17}$$

$$+\boldsymbol{F}_1(t'')\cdot\boldsymbol{\mathcal{A}}(\boldsymbol{\kappa_0}(t''))\Big]dt'' + \nabla_{\boldsymbol{K}}\varphi_d^{(j)}\Big|_{\boldsymbol{\kappa_0}(t')} a_0\left(sin(2\omega(t+\tau)) - sin(2\omega('t+\tau))\right) \tag{5.18}$$

and:

$$\boldsymbol{\kappa_0}(t) \equiv \boldsymbol{K} + a_0 sin(\omega t)\,\hat{\boldsymbol{x}} \;\; ; \;\; \boldsymbol{F}_0(t) \equiv -\frac{\partial}{\partial t}\left(a_0 sin(\omega t)\,\hat{\boldsymbol{x}}\right) \;\; ; \;\; \boldsymbol{F}_1(t) \equiv -\frac{\partial}{\partial t}\left(a_0 sin(2\omega(t+\tau))\,\hat{\boldsymbol{x}}\right) \tag{5.19}$$

The interband HHG field is deduced from the Fourier transform of the time derivative of the interband current (5.3): $\boldsymbol{E}_{HHG}(\omega) = FT\left[\frac{\partial \boldsymbol{J}_{inter}(t)}{\partial t}\right]$ with the modified semi-classical action (5.16):

$$E_{HHG}^{(i)}(\omega) = i\int_{BZ} d^3\boldsymbol{K}\int_{-\infty}^{\infty} dt \int_{-\infty}^{t} dt' F^{(j)}(t')\left|d^{(j)}(\boldsymbol{K}+\boldsymbol{A}(t'))\right|\left|d^{*(i)}(\boldsymbol{K}+\boldsymbol{A}(t))\right| e^{-i\tilde{S}^{(i)}[\boldsymbol{K},t',t,\tau]+i\omega t} + c.c \tag{5.20}$$

A tunneling event occurs along each half cycle of the laser, followed by a burst

of interband emissions, $\tilde{\boldsymbol{E}}^1_{HHG}(\omega), \tilde{\boldsymbol{E}}^2_{HHG}(\omega)$ (superscripts correspond to each half cycle), which leads to the discrete harmonic spectrum:

$$\boldsymbol{E}_{HHG}(\omega) = \sum_{N=-\infty}^{\infty} \left(\tilde{\boldsymbol{E}}^1_{HHG}(\omega) - e^{i\omega\frac{T}{2}}\tilde{\boldsymbol{E}}^2_{HHG}(\omega)\right)\delta(\omega - N\omega_0) \tag{5.21}$$

with:

$$\tilde{\boldsymbol{E}}^1_{HHG}(\omega) = i\int_{BZ} d^3\boldsymbol{K}\int_0^{\frac{T}{2}} dt\int_{-\infty}^{t} dt'\boldsymbol{F}(t')\cdot|\boldsymbol{d}(\boldsymbol{K}+\boldsymbol{A}(t'))|\,|\boldsymbol{d}^*(\boldsymbol{K}-\boldsymbol{A}(t))|\,e^{-i\tilde{S}[\boldsymbol{K},t',t,\tau]+i\omega t} + c.c = \tag{5.22}$$

$$= i\int_{BZ} d^3\boldsymbol{K}\int_0^{\frac{T}{2}} dt\int_{-\infty}^{t} dt' g^1(\boldsymbol{K},t',t)\,e^{-i\tilde{S}^1[\boldsymbol{K},t',t,\tau]+i\omega t} + c.c \tag{5.23}$$

$$\tilde{\boldsymbol{E}}^2_{HHG}(\omega) = i\int_{BZ} d^3\boldsymbol{K}\int_{\frac{T}{2}}^{T} dt\int_{-\infty}^{t} dt'\boldsymbol{F}(t')\cdot|\boldsymbol{d}(\boldsymbol{K}+\boldsymbol{A}(t'))|\,|\boldsymbol{d}^*(\boldsymbol{K}-\boldsymbol{A}(t))|\,e^{-i\tilde{S}[\boldsymbol{K},t',t,\tau]+i\omega t} + c.c = \tag{5.24}$$

$$= i\int_{BZ} d^3\boldsymbol{K}\int_0^{\frac{T}{2}} dt\int_{-\infty}^{t} dt' g^2(\boldsymbol{K},t',t)\,e^{-i\tilde{S}^2[\boldsymbol{K},t',t,\tau]+i\omega t} + c.c \tag{5.25}$$

where $g^1, \tilde{S}^1 = S_{inv} + \gamma_B^{(i)} + \epsilon\sigma^{1(i)}$ are the first half-cycle pre-exponential factors and modified action, and $g^2, \tilde{S}^2 = S_{inv} - \gamma_B^{(i)} + \epsilon\sigma^{2(i)}$ are the second half-cycle pre-exponential factors and modified action. We then demand $\nabla\tilde{S}^1 = \nabla\tilde{S}^2 = 0$ to get the SPA equations for each half-cycle (5.6) - (5.8) and find the set of stationary parameters $[K^1_{st}, {t'_{st}}^1, t^1_{st}], [K^2_{st}, {t'_{st}}^2, t^2_{st}]$ that solve them, each equal to:

$$\begin{cases} \boldsymbol{K}^a_{st} = \boldsymbol{K}^{a(0)}_{st} + \epsilon\boldsymbol{K}^{a(1)}_{st} + O(\epsilon^2) \\ {t'_{st}}^a = {t'_{st}}^{a(0)} + \epsilon{t'_{st}}^{a(1)} + O(\epsilon^2) \\ t^a_{st} = t^{a(0)}_{st} + \epsilon t^{a(1)}_{st} + O(\epsilon^2) \end{cases} \tag{5.26}$$

with $a \in \{1,2\}$ counting the half-cycles, and $\left[K^{a(0)}_{st}, {t'_{st}}^{a(0)}, t^{a(0)}_{st}\right]$ being the unperturbed saddle point solution for each half cycle. We notice that in the $\Gamma - M$ direction, since $\gamma_B^{(i)} = 0$, then the unperturbed stationary parameters are identical for both half cycles. We notice that since these solutions are the stationary points of the action, then their modification due to first order corrections vanish and

appear only in higher orders of ϵ:

$$\tilde{S}\left[\boldsymbol{K}_{st}, t'_{st}, t_{st}\right](\tau) = \tilde{S}_0\left[\boldsymbol{K}_{st}^{(0)}, {t'_{st}}^{(0)}, t_{st}^{(0)}\right] + \epsilon\sigma\left[\boldsymbol{K}_{st}, t'_{st}, t_{st}\right](\tau) + O\left(\epsilon^2\right) \tag{5.27}$$

$$\sigma\left[\boldsymbol{K}_{st}, t'_{st}, t_{st}\right](\tau) = \left.\frac{d\tilde{S}}{d\epsilon}\right|_{\epsilon=0} = \left.\frac{\partial\tilde{S}}{\partial\epsilon}\right|_{\epsilon=0} + \left.\frac{\partial\tilde{S}}{\partial t'_{st}}\frac{\partial t'_{st}}{\partial\epsilon}\right|_{\epsilon=0} + \left.\nabla_{\boldsymbol{K}_{st}}\tilde{S}\cdot\nabla_{\epsilon}\boldsymbol{K}_{st}\right|_{\epsilon=0} + \left.\frac{\partial\tilde{S}}{\partial t_{st}}\frac{\partial t_{st}}{\partial\epsilon}\right|_{\epsilon=0} \tag{5.28}$$

but since we demanded $\nabla\tilde{S} = 0$ then:

$$\left.\frac{\partial\tilde{S}}{\partial t'_{st}}\right|_{\epsilon=0} = \left.\nabla_{\boldsymbol{K}_{st}}\tilde{S}\right|_{\epsilon=0} = \left.\frac{\partial\tilde{S}}{\partial t_{st}}\right|_{\epsilon=0} = 0 \tag{5.29}$$

giving to first order in ϵ:

$$\Longrightarrow \sigma\left[\boldsymbol{K}_{st}, t'_{st}, t_{st}\right](\tau) = \sigma\left[\boldsymbol{K}_{st}^{(0)}, {t'_{st}}^{(0)}, t_{st}^{(0)}\right](\tau) \tag{5.30}$$

After applying the saddle point solutions to each half-cycle, the integrals $\int_{BZ} d^3\boldsymbol{K} \int dt \int dt'$ fall, and we get to first order in ϵ:

$$\begin{aligned}\boldsymbol{E}_{HHG}(\omega) = \sum_{N=-\infty}^{\infty} \Big(& g^1\left(\boldsymbol{K}_{st}^{1(0)}, {t'_{st}}^{1(0)}, t_{st}^{1(0)}\right) e^{-i\tilde{S}^1\left[\boldsymbol{K}_{st}^{1(0)}, {t'_{st}}^{1(0)}, t_{st}^{1(0)}\right](\tau) + i\omega t_{st}^{1(0)}} \\ & - (-1)^N g^2\left(\boldsymbol{K}_{st}^{2(0)}, {t'_{st}}^{2(0)}, t_{st}^{2(0)}\right) e^{-i\tilde{S}^2\left[\boldsymbol{K}_{st}^{2(0)}, {t'_{st}}^{2(0)}, t_{st}^{2(0)}\right](\tau) + i\omega t_{st}^{2(0)}} \Big) \delta\left(\omega - N\omega_0\right) + c.c\end{aligned}$$

We notice that if the material is inversion symmetric, then $g^1 = g^2$,$\tilde{S}^1 = \tilde{S}^2$ and the saddle point solutions for each half-cycle are identical, leading to the cancellation of even harmonics, as expected.

We would like to write the intensity of a single harmonic N:

$$\begin{aligned}I_{HHG}\left(N\omega_o\right) \propto \left|\boldsymbol{E}_{HHG}^N\right|^2 \propto & |\tilde{g}^1\left(\boldsymbol{K}_{st}^{1(0)}, {t'_{st}}^{1(0)}, t_{st}^{1(0)}, N\right) e^{-i\tilde{S}^1\left[\boldsymbol{K}_{st}^{1(0)}, {t'_{st}}^{1(0)}, t_{st}^{1(0)}\right](\tau)} \\ & \pm \tilde{g}^2\left(\boldsymbol{K}_{st}^{2(0)}, {t'_{st}}^{2(0)}, t_{st}^{2(0)}, N\right) e^{-i\tilde{S}^2\left[\boldsymbol{K}_{st}^{2(0)}, {t'_{st}}^{2(0)}, t_{st}^{2(0)}\right](\tau)}|^2\end{aligned}$$

Where '+' is for odd N and '$-$' is for even N, and now $\tilde{g}^a$ includes the $e^{iN\omega_0 t_{st}^{a(0)}}$ terms. We can write this more compactly as:

$$I_{HHG}^N(\omega) \propto \left|\alpha_1 e^{i\varphi_1} e^{i\epsilon\sigma_1(\tau)} \pm \alpha_2 e^{i\varphi_2} e^{i\epsilon\sigma_2(\tau)}\right|^2 \tag{5.31}$$

Where we decomposed $\tilde{S}$ to its constituents according to equation (5.27). We further break each of the parameters to the symmetric and anti-symmetric parts with respect to the half-cycles:

$$\alpha_1 = \alpha_0 + \Delta\alpha \quad ; \quad \alpha_2 = \alpha_0 - \Delta\alpha \tag{5.32}$$

$$\varphi_1 = \varphi_0 + \Delta\varphi \quad ; \quad \varphi_2 = \varphi_0 - \Delta\varphi \tag{5.33}$$

$$\sigma_{1,2} = \sigma^r_{1,2} - i\sigma^i_{1,2} \tag{5.34}$$

with:

$$\sigma_1^{r,i} = \sigma^{r,i} + \delta\sigma^{r,i} \tag{5.35}$$

$$\sigma_2^{r,i} = -\sigma^{r,i} + \delta\sigma^{r,i} \tag{5.36}$$

where α_0, φ_0 and $\Delta\alpha, \Delta\varphi$ are the symmetric and anti-symmetric parts of the unperturbed XUV photon amplitude and phase, representing the unperturbed ionization and recombination amplitude changes along with the action phase changes between the two half cycles. $\delta\sigma^r, \delta\sigma^i$ and σ^r, σ^i are the real and imaginary symmetric and anti-symmetric phase perturbations of the XUV photon, representing trajectory (real) and ionization (imaginary) perturbations.

We now wish to expand (5.31) to leading order in ϵ in the case of a broken inversion symmetry material (as quartz):

$$I^N_{HHG} \propto \left|\alpha_1 e^{i\varphi_1} e^{i\epsilon\sigma_1(\tau)} \pm \alpha_2 e^{i\varphi_2} e^{i\epsilon\sigma_2(\tau)}\right|^2 = |\alpha_1|^2 e^{2\epsilon\sigma_1^i} + |\alpha_2|^2 e^{2\epsilon\sigma_2^i} \pm 2\alpha_1\alpha_2 e^{\epsilon\left(\sigma_1^i+\sigma_2^i\right)} cos\left(\Delta\varphi + \epsilon\left(\sigma_1^r - \sigma_2^r\right)\right) =$$
$$|\alpha_1|^2 e^{2\epsilon\sigma_1^i} + |\alpha_2|^2 e^{2\epsilon\sigma_2^i} \pm 2\alpha_1\alpha_2 e^{\epsilon\left(\sigma_1^i+\sigma_2^i\right)} \left[cos\left(\Delta\varphi\right) cos\left(\epsilon\left(\sigma_1^r - \sigma_2^r\right)\right) \mp sin\left(\Delta\varphi\right) sin\left(\epsilon\left(\sigma_1^r - \sigma_2^r\right)\right)\right]$$

where $\Delta\varphi \equiv \varphi_1 - \varphi_2$ and in the second equality we used a trigonometric identity. The leading order is linear with ϵ:

$$I^N_{HHG} \propto |\alpha_1|^2 + |\alpha_2|^2 \pm 2\alpha_1\alpha_2 cos\left(\Delta\varphi\right) + 2|\alpha_1|^2 \epsilon\sigma_1^i + 2|\alpha_2|^2 \epsilon\sigma_2^i$$
$$\pm 2\alpha_1\alpha_2\left(\epsilon\left(\sigma_1^i + \sigma_2^i\right)\right) cos\left(\Delta\varphi\right) \mp 2\alpha_1\alpha_2 sin\left(\Delta\varphi\right)\left(\epsilon\left(\sigma_1^r - \sigma_2^r\right)\right) + O\left(\epsilon^2\right)$$

Inserting the symmetric and anti-symmetric notations from equations (5.32)-(5.36)

and after some algebra, we get:

$$I^N_{HHG} \propto 2\alpha_0^2 + 2\Delta\alpha^2 \pm 2\left(\alpha_0^2 - \Delta\alpha^2\right) cos\left(\Delta\varphi\right) + 8\alpha_0 \Delta A \epsilon \sigma^i$$
$$+ 4\left[\left(\alpha_0^2 + \Delta A^2\right) \pm \left(\alpha_0^2 - \Delta\alpha^2\right) cos\left(\Delta\varphi\right)\right] \epsilon\delta\sigma^i \mp 4\left(\alpha_0^2 - \Delta\alpha^2\right) sin\left(\Delta\varphi\right) \epsilon\sigma^r$$

The imaginary symmetric part of the perturbation $\delta\sigma^i$ can be shown to be related to the perturbation of the Berry phase under the barrier (imaginary part), which is small and thus can be neglected, finally giving:

$$I^N_{HHG} \propto 2\alpha_0^2 + 2\Delta\alpha^2 \pm 2\left(\alpha_0^2 - \Delta\alpha^2\right) cos\left(\Delta\varphi\right) + 8\alpha_0 \Delta\alpha \epsilon \sigma^i \mp 4\left(\alpha_0^2 - \Delta\alpha^2\right) sin\left(\Delta\varphi\right) \epsilon\sigma^r \tag{5.37}$$

From equation (5.37) we can see that if $\sigma^i \gg \sigma^r$ then the even and odd harmonics would be in phase, revealing the important part of the ionization and recombination in the HHG process. If we look on the difference of two adjacent even and odd harmonics (assuming similar trajectories) we get:

$$I^{diff}_{HHG}\left(\tau\right) \propto 4\left(\alpha_0^2 - \Delta\alpha^2\right) cos\left(\Delta\varphi\right) - 8\left(\alpha_0^2 - \Delta\alpha^2\right) sin\left(\Delta\varphi\right) \epsilon\sigma^r\left(\tau\right) \tag{5.38}$$

where we explicitly added the notation of the dependence of the real perturbation σ^r on the two-color delay τ. By extracting the two-color phase of I^{diff}_{HHG} as function of harmonic, we can conclude if there is a chirp in the signal, revealing the trajectory nature of the harmonics, thus favoring the interband model.

5.4 Discrete Berry phase

In the following chapter we present a general description of discrete geometrical phase and its physical manifestation as interband Berry phase. A simple way to obtain a Berry phase is to take N states $|\Psi_i\rangle$ and to consider the phase of

$$\frac{\langle\Psi_1|\Psi_2\rangle\langle\Psi_2|\Psi_3\rangle\cdots\langle\Psi_N|\Psi_1\rangle}{|\langle\Psi_1|\Psi_2\rangle\langle\Psi_2|\Psi_3\rangle\cdots\langle\Psi_N|\Psi_1\rangle|} = e^{i\gamma} \tag{5.39}$$

(for a review, see for example [41], section 2.1), as shown in Fig. 22. The phase γ is gauge invariant in the sense that it is independent of the unphysical phase of each of the states $|\Psi_i\rangle$. This discrete description is quite general, and the familiar

uses of the Berry phase are simply limits of this case.

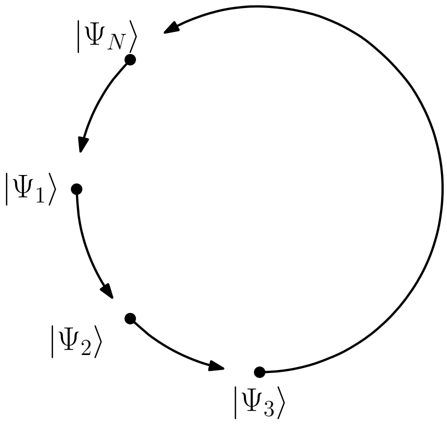

Figure 22: The accumulated Berry phase in a closed trajectory.

For example, in band theory the wavefunctions are Bloch waves $\psi_{n,k} = e^{ik\cdot r}u_{n,k}(r)$, and taking a trajectory in k space, (5.39) becomes

$$\langle u_{n,k_1}|u_{n,k_2}\rangle\langle u_{n,k_2}|u_{n,k_3}\rangle\cdots\langle u_{n,k_N}|u_{n,k_1}\rangle. \tag{5.40}$$

For nearby momenta, $\langle u_{n,k}|u_{n,k+\epsilon}\rangle \approx 1+\epsilon\langle u_{n,k}|\partial_k u_{n,k}\rangle \approx e^{\epsilon\langle u_{n,k}|\partial_k u_{n,k}\rangle}$ with $\langle u_{n,k}|\partial_k u_{n,k}\rangle$ being purely imaginary. For infinitesimal ϵ, we find

$$\langle u_{n,k_1}|u_{n,k_2}\rangle\langle u_{n,k_2}|u_{n,k_3}\rangle\cdots\langle u_{n,k_N}|u_{n,k_1}\rangle \approx e^{\epsilon\sum_i\langle u_{n,k_i}|\partial_k u_{n,k_i}\rangle} \to e^{\int dk\,\langle u_{n,k}|\partial_k u_{n,k}\rangle} \equiv e^{-i\int dk\,\mathcal{A}_n(k)} \tag{5.41}$$

which is the familiar continuum Berry phase in a band, and $\mathcal{A}_n(k)$ is the Berry connection.

Interestingly, in High Harmonic Generation, we obtain a trajectory that goes between different bands. The wavefunction starts at $\langle u_{v,k_1}|$ (the first "bra") and comes back to the same state $|u_{v,k_1}\rangle$ (the last "ket"). The *interband Berry phase* is associated with this closed loop in the energy – momentum space, with the evolution of the wavefunction including both propagation within the bands and transitions between the bands.

We can separate the loop into four parts, as shown in the Figure 23. First, the electron tunnels from the valence band to the conduction band (interband transition), associated with the term $\langle u_{v,k_1}|u_{c,k_2}\rangle$. Second, the electron propagates

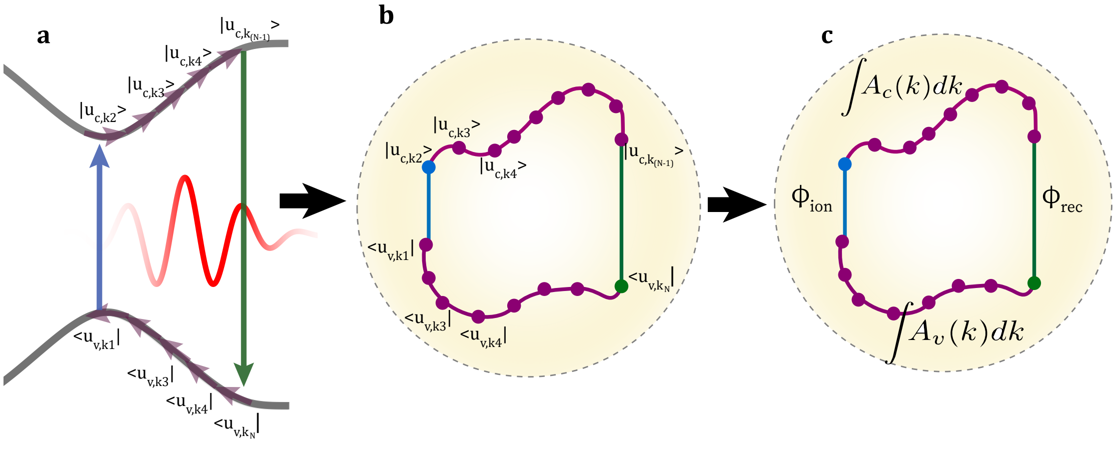

Figure 23: **Illustration of equation 1 in the main text**. (a) The evolution of the wavepcaket, along and between the bands, notated by wavefunction $|u_{n,k} > (n = v, c)$. (b) A points loop presentation for (a), where the purple line represents continuous evolution while the green and the blue lines represent the discrete evolution. (c) The associated phase for every step along the loop.

within the conduction band (intraband evolution), changing its momentum from k_2 to k_3, etc, until k_{N-1}. This evolution is described by $\langle u_{c,k_2}|u_{c,k_3}\rangle \cdot \langle u_{c,k_3}|u_{c,k_4}\rangle \cdot \langle u_{c,k_4}|u_{c,k_5}\rangle \cdots \langle u_{c,k_{N-2}}|u_{c,k_{N-1}}\rangle$. Third, the electron recombines with the hole via the interband transition, associated with $\langle u_{c,k_{N-1}}|u_{v,k_N}\rangle$. The time-reversed evolution of the hole in the bra closes the loop: the hole evolves within the valence band from $\langle u_{v,k_N}|$ back to $|u_{v,k_1}\rangle$. Figure 23 describes this evolution pictorially. The corresponding expression is:

$$\begin{aligned}
&\langle u_v(k_1)|u_c(k_i)\rangle \left[\langle u_c(k_i)|u_c(k_1)\rangle \cdots \langle u_c(k_{N-1}))|u_c(k_N)\rangle\right] \cdot \\
&\cdot \langle u_c(k_N)|u_v(k_f)\rangle \left[\langle u_v(k_f)|u_v(k_N)\rangle \cdots \langle u_v(k_2)|u_v(k_1)\rangle\right] \\
&\approx \epsilon^2 \langle \partial_k u_v(k_i)|u_c(k_i)\rangle e^{\int_{k_i}^{k_f} dk\, \langle u_c(k)|\partial_k u_c(k)\rangle} \langle u_c(k_f)|\partial_k u_v(k_f)\rangle e^{-\int_{k_i}^{k_f} dk\, \langle u_v(k)|\partial_k u_v(k)\rangle}
\end{aligned} \tag{5.42}$$

where we note that $\langle u_v(k)|u_c(k)\rangle = 0$ so that for $k_1 = k_i + \epsilon$ close to each other, we have $\langle u_v(k_1)|u_c(k_i)\rangle \approx (\langle u_v(k_i)| + \epsilon\langle \partial_k u_v(k_i)|)\,|u_c(k_i)\rangle = \epsilon\langle \partial_k u_v(k_i)|u_c(k_i)\rangle$. The continuous part of the trajectory goes from k_i to k_f. To summarize, the description of the wavefunction evolution using the Berry phase formalism (5.39)

indeed gives us the familiar High Harmonic Generation expression

$$\begin{aligned}
&\propto d^*(k_i)e^{-i\int_{k_i}^{k_f} dk\,\mathcal{A}_c(k)}d(k_f)e^{i\int_{k_i}^{k_f} dk\,\mathcal{A}_v(k)} = |d^*(k_i)||d(k_f)|e^{-i\left(\int_{k_i}^{k_f} dk\,\mathcal{A}_c(k)-\mathcal{A}_v(k)+\phi_d(k_f)-\phi_d(k_i)\right)} \\
&\gamma \equiv -\int_{k_i}^{k_f} dk\,[\mathcal{A}_c(k)-\mathcal{A}_v(k)]+\phi_d(k_f)-\phi_d(k_i)
\end{aligned} \tag{5.43}$$

This phase indeed captures the four steps of the energy- momentum loop: the phase associated with the injection (phase of the dipole at the ionization momentum, $\phi_d(k_i)$), the phase associated with the intraband evolution of the electron ($\int_{\boldsymbol{k}_i}^{\boldsymbol{k}_f} \mathcal{A}_c(\boldsymbol{k})d\boldsymbol{k}$), the phase of the recombination dipole ($\phi_d(k_f)$), and finally the intraband evolution of hole ($-\int_{\boldsymbol{k}_i}^{\boldsymbol{k}_f} \mathcal{A}_v(\boldsymbol{k})d\boldsymbol{k}$. The phase is unique due to the combination of continuous and discrete evolution where the discrete part is described by the dipole moment phase, which is a connection between the bands, similarly to the more standard connection, $\mathcal{A}_n(k)$ that goes within a band.

The discrete part of the trajectory does not allow us to write the Berry phase as a surface integral over a gauge invariant curvature in a straightforward way. This is not unusual, as there are many cases where a Berry phase cannot be written in such a way. However, we can find a quantity, that we denote by $\boldsymbol{F}(k)$, similar to the Berry curvature ($\Omega(k)$), representing the flux over an infinitesimal area, and is gauge invariant. It is given by

$$\boldsymbol{F}(\boldsymbol{k}) = \boldsymbol{\mathcal{A}}_c(k) - \boldsymbol{\mathcal{A}_v}(\boldsymbol{k}) + \nabla_k\phi_d(k). \tag{5.44}$$

It can be verified that $F(k)$ is invariant under the gauge transformation

$$\begin{aligned}
|u_c(k)\rangle &\to e^{i\alpha(k)}|u_c(k)\rangle, \\
|u_v(k)\rangle &\to e^{i\beta(k)}|u_v(k)\rangle.
\end{aligned} \tag{5.45}$$

Then, we can write the Berry phase (5.42) as a line integral over $F(k)$

$$\gamma = \int_{k_i}^{k_f} \boldsymbol{dk}\cdot\boldsymbol{F}(\boldsymbol{k}) \tag{5.46}$$

Here $\boldsymbol{F}(k)$ represents a narrow rectangle in energy-momentum space and line integration over all these rectangles gives the total surface captured by the Berry

phase, see Fig. 24.

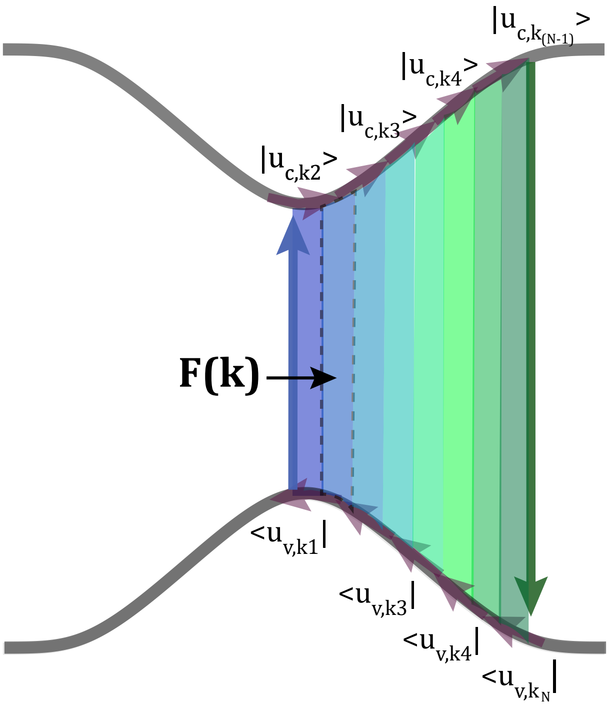

Figure 24: Integrated flux.

5.5 Differences between Berry phase and Berry curvature

Berry phase and Berry curvature are related to each other, however they lead to different phenomena in condensed matter physics [42]. The Berry curvature leads to the appearance of the anomalous velocity and the classical Hall effect. The emergence of the Berry phase leads to quantized electronic phenomena and new quantum phases of matter, such as the Quantum Hall effect and Chern insulator. Below we provide the formalism related to the Berry phase/curvature and describe how they can be resolved experimentally.

When a quantum wavefunction evolves in the parameter space, it accumulates a phase; also referred to as the Berry phase (γ_B) [42, 43]. The Berry phase is described by the integration over vector-valued function, the Berry connection ($\mathcal{A}_n(\boldsymbol{k}) = i \langle u_{n,\boldsymbol{k}}(x)| \nabla_{\boldsymbol{k}} |u_{n,\boldsymbol{k}}(x)\rangle$). The Berry connection, in every realization, is not a gauge invariant quantity and therefore it is not an observable. Unlike the Berry connection, in the particular case of a closed loop, the Berry phase becomes

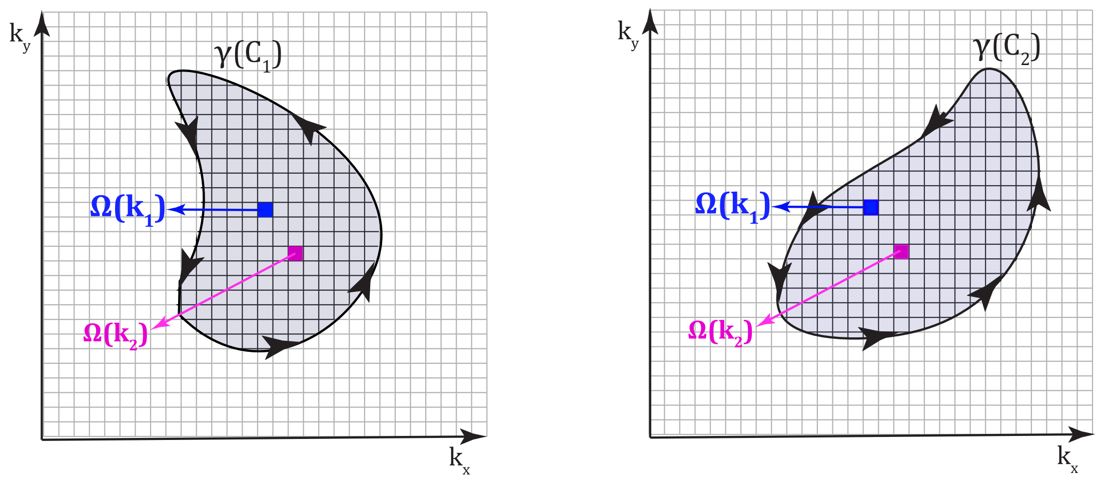

Figure 25: **Comparison between Berry phase and Berry curvature.** While the Berry curvature is defined at every point (square) in the parameter space ($\Omega(k)$, blue and pink squares), the Berry phase, $\gamma_B(C)$, depends on the closed path (black contour).

gauge invariant and can be measured. In this case, the Berry phase represents a geometrical phase; a **global** quantity that depends on the contour of the evolution only, $\gamma_B(C)$. In analogy to gauge field theories, one can define a third quantity, the Berry curvature, $\boldsymbol{\Omega}(k) = \nabla_{\boldsymbol{k}} \times \mathcal{A}$, which represents the flux inside the loop (figure 25). In contrast to the Berry phase, the Berry curvature is a **local** (rather than global) gauge invariant quantity, defined at every point in the parameter space, and can be measured even without a closed loop contour. The three quantities are related according to:

$$\gamma_B(C) = \int_C d\boldsymbol{k}\mathcal{A}(\boldsymbol{k}) = \int_S d^2\boldsymbol{k}\Omega(\boldsymbol{k}) \tag{5.47}$$

While, both the Berry phase and the Berry curvature can be measured, there are important differences between them:

1. The Berry curvature is a local property, defined at every point in k space (figure 25). Importantly, the Berry curvature does not depend on the evolution of the wavepacket. In contrast, the Berry phase depends on the contour of the evolution of the wavefunction. Therefore, in contrast to the Berry curvature, it is not a static property of the medium but a dynamical one that varies with the driving

field. Therefore, one cannot define general characteristics of the Berry phase in quartz.

2. The Berry curvature can be directly measured via the transverse polarization it induces in light matter interactions, or via transversal resistivity (Hall resistivity) in transport measurements. Such transverse polarization can be resolved in any system, as long as the inversion symmetry is broken [44].

However, resolving the Berry phase requires an interferometric scheme. Performing an interferometric measurement in condensed matter systems is extremely challenging, due to dephasing effects abundant in many-body systems. Electron-phonon, electron-electron interaction and scattering from impurities can lead to loss of the phase information. One of the most important breakthroughs of this field was accomplished by achieving very clean samples of GaAs-AlGaAs hetrostructures, establishing 2D electron gas systems at extremely low temperatures (mK scaling)[45, 46]. The coherence is achieved by fabricating these high quality devices with high mobility. Only recently, interferometry experiments in intrinsic electronic systems were performed in very clean graphene samples, at $60mK$ [47]). In contrast, in this work we resolve for the first time the Berry phase, in a trivial insulator, under room temperature conditions.

In our paper we achieve coherence by performing the interferometric measurement on an attosecond time scale, before any scattering or dephasing mechanism evolves. We believe that the ability to resolve the quantum nature of the wavefunction on extremely short time scales is one of the most important advantages of attosecond science in condensed matter systems.

6 α–quartz HHG – quantum analysis

6.1 Linearly Polarized Field

In the first stage we consider a single, multicycle field, having a linear polarization ($A^y(t) = A_0^y \sin(\omega_0 t)$). In this case, the interband mechanism leads to the emission of optical radiation every half of an optical cycle of the driving field, forming a

train of identical attosecond pulses. The emission during the positive and negative half cycles is associated with electronic trajectories driven along the positive and negative crystal momentum, respectively. Summing these two contributions coherently, can be expressed as:

$$\mathbf{E}_{HHG}(\omega) = \sum_{N=-\infty}^{\infty} i \int_{BZ} d^3K \int_{-\infty}^{t} dt' \int_{0}^{\frac{T}{2}} dt(\mathbf{F}(t') \cdot \mathbf{d}(\mathbf{K}+\mathbf{A}(t'))\mathbf{d}^*(\mathbf{K}+\mathbf{A}(t))e^{-iS[\mathbf{K},t',t]+i\omega t}$$
$$-\mathbf{F}(t') \cdot \mathbf{d}(-\mathbf{K}-\mathbf{A}(t'))\mathbf{d}^*(-\mathbf{K}-\mathbf{A}(t))e^{-iS[-\mathbf{K},t',t]+i\omega t+iN\omega_0\frac{T}{2}} + c.c)\delta(\omega - N\omega_0) \tag{6.1}$$

Where $\mathbf{E}_{HHG}$ is the harmonic field, N is the harmonic number, T and ω_0 are the fundamental field's period and frequency, respectively. According to the time reversal symmetry (TRS), **d**(-**k**) = **d***(**k**), and based on the symmetry of the generalized action, odd and even harmonic signals can be described as:

$$E^{(i)}(N_{odd}) \propto i \int_{BZ} d^3K \int_{-\infty}^{t} dt' \int_{0}^{\frac{T}{2}} dtF^{(j)}(t')|d^{(j)}(\mathbf{K}+\mathbf{A}(t'))||d^{*(i)}(\mathbf{K}+\mathbf{A}(t))|e^{-iS_{inv}[\mathbf{K},t',t]+i\omega t}$$
$$\cdot \cos(\gamma_B^{(i)}[\mathbf{K},t',t]) + c.c$$
$$E^{(i)}(N_{even}) \propto i \int_{BZ} d^3K \int_{-\infty}^{t} dt' \int_{0}^{\frac{T}{2}} dtF^{(j)}(t')|d^{(j)}(\mathbf{K}+\mathbf{A}(t'))||d^{*(i)}(\mathbf{K}+\mathbf{A}(t))|e^{-iS_{inv}[\mathbf{K},t',t]+i\omega t}$$
$$\cdot \sin(\gamma_B^{(i)}[\mathbf{K},t',t]) + c.c \tag{6.2}$$

Equation 6.2 captures the interferomatric nature of our measurements: the odd and the even harmonics encode constructive and destructive interference of the Berry phase, as their intensity presented in equation 2 at the main text.

By using the Saddle Point Approximation, we estimate the integral above according to the stationary parameters of the action ($[\mathbf{K}^{st}, t'^{st}, t^{st}]$) [12]. Driving the interaction by a linearly polarized field (along y) the intensities of odd and even harmonics can be expressed as:

$$I^{(N)} \propto \begin{cases} g_{ion} e^{-2Im(S_{inv})} \left(|d^y|^2 \left|\cos\tilde{\gamma}_B\right|^2 + |d^x|^2 \left|\cos(\tilde{\gamma}_B + \Delta\phi_d^{(x)})\right|^2 \right) & N \textit{ is odd} \\ g_{ion} e^{-2Im(S_{inv})} \left(|d^y|^2 \left|\sin\tilde{\gamma}_B\right|^2 + |d^x|^2 \left|\sin(\tilde{\gamma}_B + \Delta\phi_d^{(x)})\right|^2 \right) & N \textit{ is even} \end{cases} \tag{6.3}$$

Where g_{ion} is the ionization probability, determined by the field's amplitude and the dipole coupling at the stationary tunneling time and momentum. d^x, d^y are the recombination dipole components, estimated by the stationary recombination time and momentum. Finally, S_{inv} and $\tilde{\gamma}_B$ are determined at the stationary parameters as well (note that these are complex quantities)

In the following we focus on the $\Gamma - M$ and $\Gamma - K$ symmetry axes. Quartz has a C_2 symmetry along the $\Gamma - K$ direction. Therefore, when the laser is polarized along the $\Gamma - M$ direction (i.e in perpendicular to the C_2 symmetry axis), the dipoles will transform as:

$$\begin{cases} d^x_{mn}\left(-k_x, k_y\right) = s_m s_n d^x_{mn}\left(k_x, k_y\right) \\ d^y_{mn}\left(-k_x, k_y\right) = -s_m s_n d^y_{mn}\left(k_x, k_y\right) \end{cases} \tag{6.4}$$

where $s_m, s_n \in \{\pm 1\}$ are the parity eigenvalues of the Bloch wavefunction [48, 49]. For the first conduction band and the highest coupled valence band, denoted c and v, it holds that $s_c = s_v = 1$. In this case, d^y_{cv} is purely imaginary ($\phi_d^{(y)} = \frac{\pi}{2}$) and d^x_{cv} is purely real ($\phi_d^{(x)} = 0$) , for all crystal momentum values, concluding that $\Delta\phi_d^{(x)} = \frac{\pi}{2}$. Such symmetries results in:

$$I^{(N)} \propto \begin{cases} e^{-2Im(S_{inv})} \left(|d^y|^2 \left|\cos\tilde{\gamma}_B\right|^2 + |d^x|^2 \left|\sin\tilde{\gamma}_B\right|^2 \right) & N \textit{ is odd} \\ e^{-2Im(S_{inv})} \left(|d^y|^2 \left|\sin\tilde{\gamma}_B\right|^2 + |d^x|^2 \left|\cos\tilde{\gamma}_B\right|^2 \right) & N \textit{ is even} \end{cases} \tag{6.5}$$

Additionally, along the $\Gamma - M$ direction, it holds that $\mathcal{A}^y\left(-k_y, 0\right) = -\mathcal{A}^y\left(k_y, 0\right)$ due to the C_2 symmetry in the perpendicular direction. In addition, $\boldsymbol{\mathcal{A}}_g\left(-\boldsymbol{k}\right) = \boldsymbol{\mathcal{A}}_g\left(\boldsymbol{k}\right)$ due to TRS. Combining the two symmetries, we conclude that along this direction $\mathcal{A}^y = 0$ and therefore $\tilde{\gamma}_B = 0$. Eq. 6.5 is therefore reduced to:

$$I^{(N)} \propto \begin{cases} e^{-2Im(S_{inv})} \left|d^y\right|^2 & N \textit{ is odd} \\ e^{-2Im(S_{inv})} \left|d^x\right|^2 & N \textit{ is even} \end{cases} \tag{6.6}$$

In contrast, when the field is linearly polarized along the $\Gamma - K$ direction, it holds that $d^x = 0$ [7], therefore relation 6.3 becomes:

$$I^{(N)} \propto \begin{cases} e^{-2Im(S_{inv})}\left(|d^y|^2 \left|\cos\tilde{\gamma}_B\right|^2\right) & N \text{ is odd} \\ e^{-2Im(S_{inv})}\left(|d^y|^2 \left|\sin\tilde{\gamma}_B\right|^2\right) & N \text{ is even} \end{cases} \tag{6.7}$$

6.2 Elliptically Polarized Field

In this chapter we study HHG spectroscopy using elliptically polarized light, $\boldsymbol{A}(t) = A_0\left(\epsilon cos\left(\omega_0 t\right), sin\left(\omega_0 t\right)\right)$, while controlling the ellipticity degree of the light, ϵ. As in the case of linear polarization, the instantaneous field reverses the order between two consecutive half cycles, therefore the temporal symmetry is preserved and eq. (6.3) remains valid. We focus on the perturbative limit, $\epsilon << 1$, where the main parameters of the interaction are unchanged, while the ellipticity of the field induces a small lateral drift. Specifically, we use the unperturbed stationary parameters along the main polarization axis, $(K_y^{st}, t'^{st}, t^{st})$, adding the transversal evolution by ϵK_x^{st}, $(\epsilon K_x^{st}, K_y^{st}, t'^{st}, t^{st})$. We expand the action with ϵ as:

$$S^{(i)}[\mathbf{K}, t', t] \approx S_0^{(i)} + \frac{\partial S^{(i)}}{\partial \epsilon}\epsilon = S_{inv,0} + \gamma_{B,0}^{(i)} + \frac{\partial S_{inv}}{\partial \epsilon}\epsilon + \frac{\partial \gamma_B^{(i)}}{\partial \epsilon}\epsilon + ... \tag{6.8}$$

Where $S_{inv,0}$ and $\gamma_{B,0}^{(i)}$ represent the unperturbed action for linear polarized field. We focus on the main axes, $\Gamma - K$ and $\Gamma - M$. First, we analyse the symmetric part of the action, S_{inv}. Its dependence on ϵ can be expressed as: $\frac{\partial S_{inv}}{\partial \epsilon} = \frac{\partial S_{inv}}{\partial K_x} K_x^{st}$. Along the main axes, the first order term in the expansion vanishes, therefore only second order terms contribute. We define the ellipticity response of this term as $\Delta S_{inv} \equiv \Delta \varepsilon_g \equiv \frac{\partial S_{inv}}{\partial^2 K_x^{st}} \epsilon^2 {K_x}^2$ (see in the main text). However, the Berry phase leads to a first order contribution, dictated by the transverse crystal momentum as well as the transverse field:

$$\frac{\partial \gamma_B^{(i)}}{\partial \epsilon} \approx \frac{\partial \tilde{\gamma}_B}{\partial \epsilon} \propto \int_{t'}^{t} [\frac{\partial (F^{(y)} \mathcal{A}_g^{(y)})}{\partial \epsilon} + \frac{\partial (F^{(x)} \mathcal{A}_g^{(x)})}{\partial \epsilon} + \frac{\partial (F^{(y)} \nabla_K \phi_d^{(y)})}{\partial \epsilon}] d\tau = \\ \int_{t'}^{t} [F^{(y)} \frac{\partial \mathcal{A}_g^{(y)}}{\partial K_x} (K_x^{st} - A_x(\tau)) + (\mathcal{A}_g^{(x)} F^{(x)}) + F^{(y)} \frac{\partial \nabla_K \phi_d^{(y)}}{\partial K_x} (K_x^{st} - A_x(\tau))] d\tau \tag{6.9}$$

Along $\Gamma - K$, the C_2 symmetry dictates two conditions: $\mathcal{A}_g^{(x)} = 0$ and $\frac{\partial \mathcal{A}_g^{(y)}}{\partial K_x} =$

$\frac{\partial \nabla_K \phi_d^{(y)}}{\partial K_x} = 0$. Therefore, along this axis, all first order ϵ-dependent terms vanish. However, along $\Gamma - M$ these three terms contribute to the *first order* perturbation. This perturbation is captured by the additional Berry phase, defined in the main text as $\Delta\gamma_B$ ($\Delta\gamma_B = \frac{\partial \gamma_B^{(i)}}{\partial \epsilon}\epsilon$). Note that this phase is accumulated along the entire trajectory and therefore is dominated by the time integrated terms $\tilde{\gamma}_B$.

Along the $\Gamma - M$ axis, the perturbation in the semiclassical action modifies the emitted field according to: $E \approx E_0 \exp(i\Delta\gamma_B + i\Delta\varepsilon_g)$, where E_0 represents the unperturbed field. The perturbation of two consecutive half-cycles is mapped onto the harmonics spectrum. Odd harmonics represent constructive interference between the fields emitted along the positive and negative half cycles, while even harmonics represent their destructive interference:

$$E_{even}^{odd}(\epsilon) = E^+(\epsilon) \pm E^-(\epsilon) \approx [E_0^+ \exp(i\Delta\gamma_B^+ + i\Delta\varepsilon_g^+) \pm E_0^- \exp(i\Delta\gamma_B^- + i\Delta\varepsilon_g^-)] \quad (6.10)$$

where $\Delta\gamma_B^\pm$, $\Delta\varepsilon_g^\pm$ represent the perturbation along the first and second half cycle. Along $\Gamma - M$, $\Delta\gamma_B^+ = -\Delta\gamma_B^- \equiv \Delta\gamma_B$, and $\Delta\varepsilon_g^+ = \Delta\varepsilon_g^- \equiv \Delta\varepsilon_g$. Therefore:

$I_{even}^{odd}(\epsilon) \propto e^{-2\Im(\Delta\varepsilon_g)} |E_0^+ \exp(i\Delta\gamma_B) \pm E_0^- \exp(-i\Delta\gamma_B)|^2$ (equation 2 in the main text). Considering the contribution of the recombination dipole moments and their vectorial properties (equations 6.4, 6.5) to the unperturbed fields, we can write the ellipticity dependence of the odd and the even harmonics intensities as:

$$I^{(N)} \propto \begin{cases} Ce^{-2Im(\Delta S_{inv})}\left(|d^y|^2 |\cos(\Delta\gamma_B(\epsilon))|^2 + |d^x|^2 |\sin(\Delta\gamma_B(\epsilon))|^2\right) & N \textit{ is odd} \\ Ce^{-2Im(\Delta S_{inv})}\left(|d^y|^2 |\sin(\Delta\gamma_B(\epsilon))|^2 + |d^x|^2 |\cos(\Delta\gamma_B(\epsilon))|^2\right) & N \textit{ is even} \end{cases} \quad (6.11)$$

Where C represents all unperturbed prefactors.

6.2.1 Reconstruction of the Berry Phase

In the following section we describe the reconstruction procedure of the Berry phase accumulated along the trajectory, shaped by the elliptical field along $\Gamma - M$ (equation (6.11)). The signal of the odd and even harmonics provide two different measurements of the interferometer. In the first stage we combine neighboring odd and even harmonics according to:

$$I^{+}_{\Gamma-M}(\epsilon) = \frac{I^{odd}_{\Gamma-M}(\epsilon) + I^{even}_{\Gamma-M}(\epsilon)}{I^{odd}_{\Gamma-M}(0)} = f(\epsilon)(1+r)\cosh\left(2Im(\Delta\gamma_B(\epsilon))\right) \tag{6.12}$$

$$I^{-}_{\Gamma-M}(\epsilon) = \frac{I^{odd}_{\Gamma-M}(\epsilon) - I^{even}_{\Gamma-M}(\epsilon)}{I^{odd}_{\Gamma-M}(0)} = f(\epsilon)(1-r)\cos\left(2Re(\Delta\gamma_B(\epsilon))\right) \tag{6.13}$$

where $r = \frac{|d_x|^2}{|d_y|^2}$ is the dipole polarization ratio, and $f(\epsilon) = e^{-2Im(\Delta S_{inv}(\epsilon))}$ is the symmetric ellipticity coefficient. This coefficient represents the suppression of the harmonic signal with the ellipticity of the driving field. Here we assume that the dipoles, their amplitudes and phases, are not modified by the elliptical perturbation. We determine their values by the measurement at $\epsilon = 0$. Under these conditions, the dipole polarization ratio is the ratio between odd and even intensities (equation (6.6)), $r = \frac{I^{even}_{\Gamma-M}(0)}{I^{odd}_{\Gamma-M}(0)}$.

We extract $f(\epsilon)$ by resolving the ellipticity response along $\Gamma - K$. The ellipticity response along this axis can be expressed as:

$$I^{+}_{\Gamma-K}(\epsilon) = \frac{I^{odd}_{\Gamma-K}(\epsilon) + I^{even}_{\Gamma-K}(\epsilon)}{I^{odd}_{\Gamma-K}(0) + I^{even}_{\Gamma-K}(0)} = f_{\Gamma-K}(\epsilon) \tag{6.14}$$

We note that this coefficient can be different along the two symmetry axes, $\Gamma - K$ and $\Gamma - M$, since it depends on the band dispersion. We correct for this difference using the two-color measurements (see next chapter for further details). Our experimental results show that the signal decay with ellipticity along $\Gamma - M$ is broader by 30% compared to $\Gamma - K$. Therefore, we consider such difference by taking: $f_{\Gamma-M} = 0.7 \cdot f_{\Gamma-K}$.

6.2.2 Theoretical Berry phase calculation

We perform a theoretical calculation of the interband Berry phase accumulated by the electron-hole wavepacket during a half cycle along the $\Gamma - M$ direction. We start with a density-functional theory (DFT) calculation of the α-quartz band structure and extract Wannier functions. From the the Wannier function-based Hamiltonian, we derive the Berry curvature and Berry connection.We focus on the

band gap between an inner valence band (such to avoid level crossings which affect the numerics) and lowest conduction band $(10.7 - 12.1eV)$. In the next step we perform a semi-classical analysis, which calculates the k-space trajectory for each ionization time, for various values of the field's ellipticity. The calculations are performed for field intensity of $0.007\,[a.u]$, wavelength of $\lambda = 1.2\,[\mu m]$, for 21 different ionization times. We then numerically calculate the interband Berry phase acquired by the electron-hole wavepacket using the k-space trajectory, according to the following equation:

$$\gamma_{B,inter}\left(t', \epsilon\right) = -\int_{\boldsymbol{k}^{\epsilon}_{t'}(t')}^{\boldsymbol{k}^{\epsilon}_{t'}(t)} \boldsymbol{\mathcal{A}}_g\left(\boldsymbol{k}\right) \cdot d\boldsymbol{k} + \phi^y_d\left(\boldsymbol{k}^{\epsilon}_{t'}\left(t\right)\right) - \phi^y_d\left(\boldsymbol{k}^{\epsilon}_{t'}\left(t'\right)\right) \tag{6.15}$$

Where $\boldsymbol{\mathcal{A}}_g\left(\boldsymbol{k}\right) = \boldsymbol{\mathcal{A}}_c\left(\boldsymbol{k}\right) - \boldsymbol{\mathcal{A}}_v\left(\boldsymbol{k}\right)$ is the conduction-valence Berry connection difference, and ϕ^y_d is the conduction-valence dipole phase along the ionization direction. Equation (6.15) provides the gauge invariant loop through crystal momentum-energy space that introduces the interband Berry phase. In this scheme an electron is ionized at t', accumulating the ionization dipole phase $\phi^y_d\left(\boldsymbol{k}^{\epsilon}_{t'}\left(t'\right)\right)$, then the electron and hole propagate, each one in their band, accumulating the Berry connection at each k-point, until finally at time t they recombine, accumulating the recombination dipole phase $\phi^y_d\left(\boldsymbol{k}^{\epsilon}_{t'}\left(t\right)\right)$ and closing the loop in parameter space.

Once we calculate the interband Berry phase for each ionization time, we map the recombination energies of each trajectory to its emission energy. We consider the finite bandwidth of each harmonic by averaging the phase over ionization times associated with an energy window of $\pm 0.1\,[eV]$. We perform this procedure for various values of the field's ellipticity. Finally, in the experiment we are sensitive to the anti-symmetric part of the interband Berry phase, only. Therefore, we anti-symmetrise our results, and take their absolute value.
Since our calculation is limited to the first band gap, we focus on studying the Berry phase dependence on the recombination energy (can be mapped to the photon energy in the HHG spectrum). Here each recombination energy represents a different trajectory in momentum-energy space. Using this calculation we can visualize how the accumulated berry phase follows the wavepacket k-space evolution.

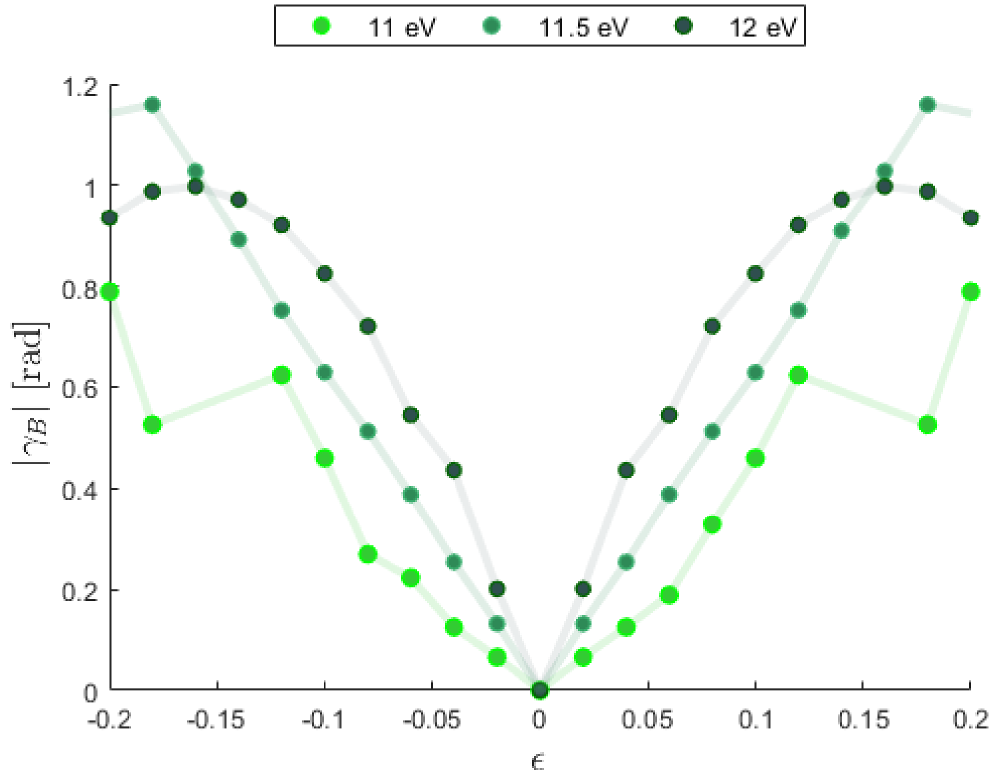

Figure 26: Interband Berry phase for trajectories associated with different recombination energies.

Figure 26 shows the accumulated interband Berry phase for the roughly shortest trajectory (corresponds to $11eV$ recombination energy) and the longest trajectory ($12eV$). As can be seen by our calculation, as the trajectory length increases– the accumulated Berry phase is larger. This validates our reconstruction results where the reconstructed Berry phase increases with harmonic order (figure 2 in the main text). Finally, the calculated Berry phase at high ellipticity values ranges between $0.8 - 1.2$ $[rad]$ which is close to the reconstructed Berry phase of harmonics 14-15. This is due to the similar trajectory length associated with these these harmonics.

6.3 Two-color Field

We observe the role of the Berry curvature by applying a driving field with a mirror symmetry between the two consecutive half cycles. In this case, the induced trans-

verse perturbation is symmetric (figure 3b in the main text): $(k_x, k_y) \to (k_x, -k_y)$. Therefore, in strong contrast to the elliptically polarized field case (chapter 6.2), the two color field does not induce a phase difference between the two interfering trajectories, associated with the Berry phase. Following equation (6.9), one can see that the first order perturbation is symmetric with respect to the two half-cycles:

$$[F^{(y)}(t)\frac{\partial \mathcal{A}_g^{(y)}}{\partial K_x}]_{K_x=0,K_y}(K_x - A_x(\tau)) = [F^{(y)}(t+\frac{T}{2})\frac{\partial \mathcal{A}_g^{(y)}}{\partial K_x}]_{K_x=0,-K_y}(K_x - A_x(\tau)) =$$
$$(\text{-}1)^2 F^{(y)}(t)\frac{\partial \mathcal{A}_g^{(y)}}{\partial K_x}{}_{K_x=0,K_y}(K_x - A_x(\tau))$$
$$[\mathcal{A}_g^{(x)}]_{K_x=0,K_y} F^{(x)}(t) = [\mathcal{A}_g^{(x)}]_{K_x=0,-K_y} F^{(x)}(t+\frac{T}{2}) \tag{6.16}$$

Our experimental results confirm the absence of a phase difference, showing a similar response of the odd and the even harmonics, with respect to the two color delay. However, due to the symmetry of the driving field, the effect of the Berry curvature on the wavepackets evolution is not canceled out and dominates the harmonic signal.

Using the semi-classical equation, we can write the evolution of the electron-hole wavepacket in real space and extract their recombination condition (chapter 4). For linearly polarized driving field (y direction), along the main axes of the crystal, the 2D wavepacket evolution is described by:

$$\begin{aligned}
\dot{k}_y &= -A_0^y \sin(\omega_0 t) \\
\dot{k}_x &= 0 \\
\Delta y(t', t) &= \int_{t'}^{t} \frac{\partial \varepsilon_g(k_x, k_y(\tau))}{\partial k_y} d\tau \\
\Delta x(t', t) &= -\int_{t'}^{t} F_0^y \cos(\omega_0 \tau) \Omega_g^z(k_x, k_y(\tau)) d\tau
\end{aligned} \tag{6.17}$$

Consider t' as the ionization time, the relative displacement of the hole and the electron at a given time t is $\Delta x, \Delta y$. The recombination condition, which dictates the recombination time t is $\Delta x = \Delta y = 0$.

According to Time Reversal Symmetry, $\varepsilon(-\mathbf{k}) = \varepsilon(\mathbf{k}), \mathbf{\Omega}(-\mathbf{k}) = -\mathbf{\Omega}(\mathbf{k})$, the evolution along the y direction is reversed during the two subcycles of the driving field,

while along the x direction it remains the same. Therefore, in the presence of a Berry curvature, the wavepackets evolution along the two half cycles are mirror images of each other (as presented in figure 3 in the main text and in chapter 4.2). Adding a weak second harmonic field (SH), orthogonally polarized with respect to the fundamental field, $\epsilon A_0^y \sin(2\omega_0 t + \varphi)$, induces an additional evolution along the transverse direction:

$$\begin{aligned} \dot{k}_x &= -\epsilon A_0^y \sin(2\omega_0 t + \varphi) \\ \Delta x(t', t, \varphi) &= \int_{t'}^{t} \frac{\partial \varepsilon_g(k_x(\tau,\varphi), k_y(\tau))}{\partial k_x} - F_0^y \cos(\omega_0 \tau) \Omega_g^z(k_x(\tau,\varphi), k_y(\tau)) d\tau \end{aligned} \tag{6.18}$$

The SH field contribution to the transverse displacement depends on φ, the delay between the two fields. Therefore, by scanning this delay, we can compensate or enhance the transverse displacement caused by the original anomalous velocity. Since the HHG signal is extremely sensitive to the recombination probability, this manipulation is mapped directly to the harmonic intensities [50]. Note that here we assume that due to the perturbative nature of the second harmonic field, the ionization time, recombination times and the y direction propagation are not modified, being dictated by the fundamental field only.

In order to take a deeper insight, we study the dependence of the harmonic signal on the two color delay. As was previously studied in gas phase HHG spectroscopy, the harmonic intensity depends exponentially on the recombination displacement, $exp^{-C|\Delta_x|^2}$ (where C is a constant), being insensitive to its sign [51]. Therefore, in absence of the anomalous velocity, where the Berry curvature is zero, the harmonic signal oscillates at an even integer of the SH frequency:

$$\begin{aligned} |\Delta x(\varphi)|^2 &= \int_{t'}^{t} \frac{\partial \varepsilon_g(k_x(\tau,\varphi), k_y(\tau))}{\partial k_x} d\tau \\ &= |\int_{t'}^{t} (a_1 k_x(\tau,\varphi) + a_3 (k_x(\tau,\varphi))^3 + ...) d\tau|^2 \propto b_2 \sin(2\varphi + \varphi_2) + b_4 \sin(4\varphi + \varphi_4)... \end{aligned} \tag{6.19}$$

Where $a_1, a_3 ..., b_2, b_4, ...$ and $\varphi_2, \varphi_4, ...$ are constants. However, along crystal orientations associated with non-vanishing Berry curvature, the anomalous velocity contribution includes a term that is independent of the two color delay. This can

be obtain by expanding the anomalous velocity contribution up to the first order in ϵ, $F_0^y \cos(\omega_0 \tau)[\frac{\partial \Omega_g}{\partial k_x}(k_x(\tau,\varphi)) + \Omega_g(k_y(\tau))]$, reducing the oscillation frequency of the harmonic signal:

$$|\Delta x(\varphi)|^2 = |\int_{t'}^{t} (a_1 k_x(\tau,\varphi) + a_3(k_x(\tau,\varphi))^3 + ... + F_0^y \cos(\omega_0 \tau)[\Omega_g(k_y(\tau)) + \frac{\partial \Omega_g}{\partial k_x}(k_x(\tau,\varphi))])d\tau|^2$$
$$\propto c_0 + c_1 \sin(\varphi + \varphi_1) + c_2 \sin(2\varphi + \varphi_2) ... \tag{6.20}$$

Where $c_0, c_1, ...$ are constants and proportional to the Berry curvature and the field amplitude $c_0 \propto F_0^y \Omega_g$. This simple picture is reflected in our experimental results, presented in figure 3 in the main text.

6.3.1 Theoretical study

To support the experimental findings in Fig.3c (see main text), we performed numerical calculations on a two-band gapped-graphene model [52], which captures the essential physics discussed in the manuscript. The most notable differences are that the minimum band gap for gapped graphene is at the K and K' points, as opposed to the Γ point in α quartz, and that the mirror symmetry direction is along Γ-M for gapped graphene, while it is along Γ-K for α quartz. The Berry curvature of gapped graphene is shown in Fig. 27a). We chose the minimum band gap of the model system equal to that reported for α quartz (9 ev), and used similar laser parameters to those estimated for the experiment: a linearly-polarized 1.2 μm and its perpendicular second harmonic, with intensities of 30 TW/cm^2 and 0.08 TW/cm^2, respectively.

We performed calculations for three different polarization angles with respect to the sample: 0° (Γ-M), 5° and 30°. For each polarization angle, we performed a time delay scan between the ω and 2ω pulses on the range $[0, \frac{2\pi}{2\omega}]$, and extracted the integrated harmonic yield for each delay. Fig. 27b-d show the oscillation of the harmonic yield as a function of the two-color time delay for the three different polarization angles.

When the laser is polarized along the mirror symmetry direction (Fig. 27b,

to be compared with the 0° panel in Fig.27c), the harmonic yield beats with the characteristic 4ω periodicity of ω-2ω experiments in inversion symmetric targets, as explained in the main text and as observed in Fig.3c. As the polarization direction is moved away from the mirror symmetry axis, the period reduces to one cycle of the 2ω field (Fig. 27c,d, to be compared with the 5° and 30° panels of Fig.27c, respectively), as explained in the main text.

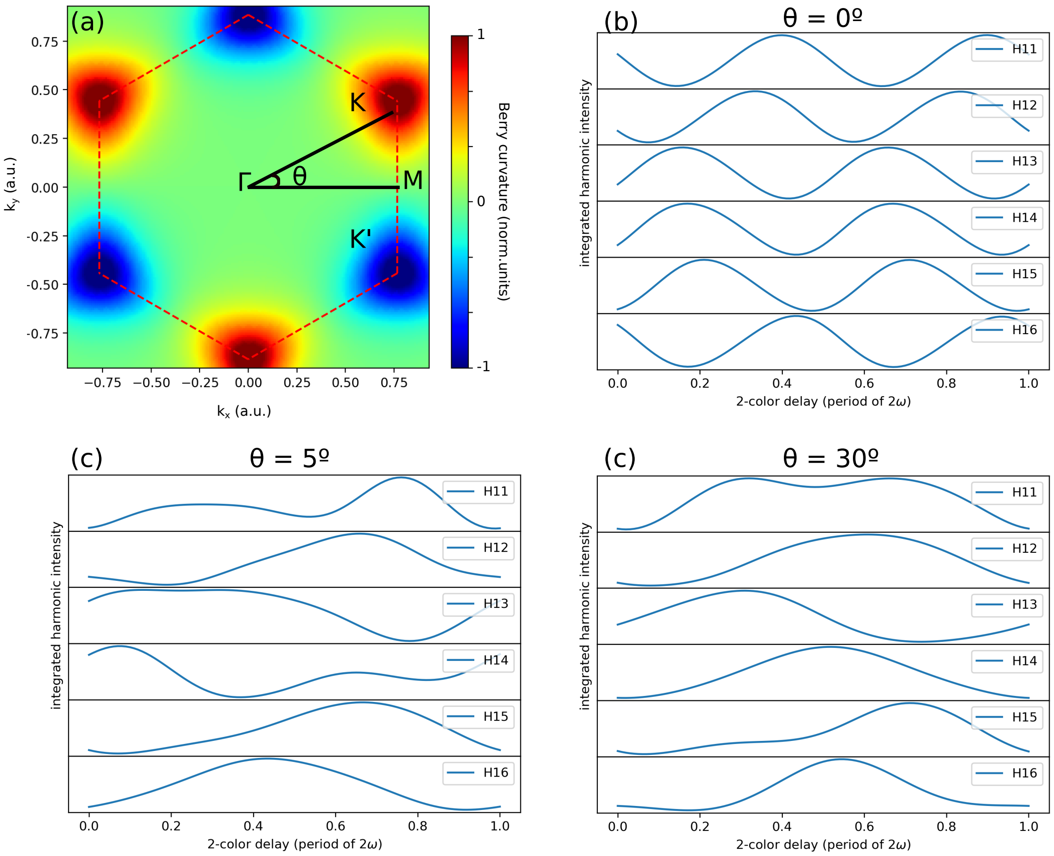

Figure 27: Two-color numerical simulations for a gapped-graphene model. (a) Berry curvature in the first Brillouin zone (red-dashed hexagon) of the conduction band. (b-d) Integrated yield of harmonics 11 to 16 as a function of the two-color time delay, for polarization directions along $\theta = 0°$ (b), $\theta = 5°$ (c) and $\theta = 30°$ (d).

References

[1] Georg Kresse and Jürgen Furthmüller. Efficiency of ab-initio total energy calculations for metals and semiconductors using a plane-wave basis set. *Computational materials science*, 6(1):15–50, 1996.

[2] Georg Kresse and Jürgen Furthmüller. Efficient iterative schemes for ab initio total-energy calculations using a plane-wave basis set. *Physical Review B*, 54(16):11169, 1996.

[3] John P Perdew, Kieron Burke, and Matthias Ernzerhof. Generalized gradient approximation made simple. *Physical Review Letters*, 77(18):3865, 1996.

[4] Peter E Blöchl. Projector augmented-wave method. *Physical Review B*, 50(24):17953, 1994.

[5] Georg Kresse and Daniel Joubert. From ultrasoft pseudopotentials to the projector augmented-wave method. *Physical Review B*, 59(3):1758, 1999.

[6] Giovanni Pizzi, Valerio Vitale, Ryotaro Arita, Stefan Blügel, Frank Freimuth, Guillaume Géranton, Marco Gibertini, Dominik Gresch, Charles Johnson, Takashi Koretsune, et al. Wannier90 as a community code: new features and applications. *Journal of Physics: Condensed Matter*, 32(16):165902, 2020.

[7] Tran Trung Luu and Hans Jakob Wörner. Measurement of the Berry curvature of solids using high-harmonic spectroscopy. *Nat Commun*, 9(1):916, December 2018.

[8] Hanzhe Liu, Yilei Li, Yong Sing You, Shambhu Ghimire, Tony F Heinz, and David A Reis. High-harmonic generation from an atomically thin semiconductor. *Nature Physics*, 13(3):262, 2017.

[9] Manish Garg, Minjie Zhan, Tran Trung Luu, H Lakhotia, Till Klostermann, Alexander Guggenmos, and Eleftherios Goulielmakis. Multi-petahertz electronic metrology. *Nature*, 538(7625):359, 2016.

[10] Shambhu Ghimire, Anthony D DiChiara, Emily Sistrunk, Pierre Agostini, Louis F DiMauro, and David A Reis. Observation of high-order harmonic generation in a bulk crystal. *Nature physics*, 7(2):138, 2011.

[11] Tran Trung Luu, M Garg, S Yu Kruchinin, Antoine Moulet, M Th Hassan, and Eleftherios Goulielmakis. Extreme ultraviolet high-harmonic spectroscopy of solids. *Nature*, 521(7553):498, 2015.

[12] G Vampa, TJ Hammond, N Thiré, BE Schmidt, F Légaré, CR McDonald, T Brabec, and PB Corkum. Linking high harmonics from gases and solids. *Nature*, 522(7557):462, 2015.

[13] Manish Garg, Hee-Yong Kim, and Eleftherios Goulielmakis. Ultimate waveform reproducibility of extreme-ultraviolet pulses by high-harmonic generation in quartz. *Nature Photonics*, 12(5):291–296, 2018.

[14] TJ Hammond, DM Villeneuve, and PB Corkum. Producing and controlling half-cycle near-infrared electric-field transients. *Optica*, 4(7):826–830, 2017.

[15] Yong Sing You, Yanchun Yin, Yi Wu, Andrew Chew, Xiaoming Ren, Fengjiang Zhuang, Shima Gholam-Mirzaei, Michael Chini, Zenghu Chang, and Shambhu Ghimire. High-harmonic generation in amorphous solids. *Nature communications*, 8(1):724, 2017.

[16] Nicola Marzari, Arash A Mostofi, Jonathan R Yates, Ivo Souza, and David Vanderbilt. Maximally localized wannier functions: Theory and applications. *Reviews of Modern Physics*, 84(4):1419, 2012.

[17] REF Silva, F Martín, and M Ivanov. High harmonic generation in crystals using maximally localized wannier functions. *Physical Review B*, 100(19):195201, 2019.

[18] EI Blount. Formalisms of band theory. In *Solid state physics*, volume 13, pages 305–373. Elsevier, 1962.

[19] N Dudovich, Olga Smirnova, J Levesque, Yu Mairesse, M Yu Ivanov, DM Villeneuve, and Paul B Corkum. Measuring and controlling the birth of attosecond xuv pulses. *Nature physics*, 2(11):781, 2006.

[20] O Pedatzur, G Orenstein, V Serbinenko, H Soifer, BD Bruner, AJ Uzan, DS Brambila, AG Harvey, L Torlina, F Morales, O Smirnova, and N Dudovich. Attosecond tunnelling interferometry. *Nature Physics*, 11(10):815, 2015.

[21] Ayelet J Uzan, Hadas Soifer, Oren Pedatzur, Alex Clergerie, Sylvain Larroque, Barry D Bruner, Bernard Pons, Misha Ivanov, Olga Smirnova, and Nirit Dudovich. Spatial molecular interferometry via multidimensional high-harmonic spectroscopy. *Nature Photonics*, pages 1–7, 2020.

[22] D Shafir, Y Mairesse, DM Villeneuve, PB Corkum, and N Dudovich. Atomic wavefunctions probed through strong-field light-matter interaction. *Nature Physics*, 5(6):412, 2009.

[23] Ayelet Julie Uzan, Gal Orenstein, Álvaro Jiménez-Galán, Chris McDonald, Rui EF Silva, Barry D Bruner, Nikolai D Klimkin, Valerie Blanchet, Talya Arusi-Parpar, Michael Krüger, et al. Attosecond spectral singularities in solid-state high-harmonic generation. *Nature Photonics*, 14(3):183–187, 2020.

[24] Giulio Vampa, Jian Lu, Yong Sing You, Denitsa R Baykusheva, Mengxi Wu, Hanzhe Liu, Kenneth J Schafer, Mette B Gaarde, David A Reis, and Shambhu Ghimire. Attosecond synchronization of extreme ultraviolet high harmonics from crystals. *Journal of Physics B: Atomic, Molecular and Optical Physics*, 53(14):144003, 2020.

[25] Yong Sing You, Mengxi Wu, Yanchun Yin, Andrew Chew, Xiaoming Ren, Shima Gholam-Mirzaei, Dana A Browne, Michael Chini, Zenghu Chang, Kenneth J Schafer, et al. Laser waveform control of extreme ultraviolet high harmonics from solids. *Optics letters*, 42(9):1816–1819, 2017.

[26] Ayelet J Uzan-Narovlansky, Álvaro Jiménez-Galán, Gal Orenstein, Rui EF Silva, Talya Arusi-Parpar, Sergei Shames, Barry D Bruner, Binghai Yan, Olga Smirnova, Misha Ivanov, et al. Observation of light-driven band structure via multiband high-harmonic spectroscopy. *Nature Photonics*, 16(6):428–432, 2022.

[27] Gal Orenstein, Oren Pedatzur, Ayelet J Uzan, Barry D Bruner, Yann Mairesse, and Nirit Dudovich. Isolating strong-field dynamics in molecular systems. *Physical Review A*, 95(5):051401, 2017.

[28] Doron Azoury, Omer Kneller, Shaked Rozen, Barry D Bruner, Alex Clergerie, Yann Mairesse, Baptiste Fabre, Bernard Pons, Nirit Dudovich, and Michael Krüger. Electronic wavefunctions probed by all-optical attosecond interferometry. *Nature Photonics*, 13(1):54–59, 2019.

[29] Mengxi Wu, Shambhu Ghimire, David A. Reis, Kenneth J. Schafer, and Mette B. Gaarde. High-harmonic generation from bloch electrons in solids. *Phys. Rev. A*, 91:043839, Apr 2015.

[30] W. Kohn. Analytic properties of bloch waves and wannier functions. *Phys. Rev.*, 115:809–821, Aug 1959.

[31] Graham G. Brown, Álvaro Jiménez-Galán, Rui E. F. Silva, and Misha Ivanov. A real-space perspective on dephasing in solid-state high harmonic generation, 2022.

[32] José D. Secada. Numerical evaluation of the hankel transform. *Computer Physics Communications*, 116(2):278–294, 1999.

[33] Mengxi Wu, Dana A. Browne, Kenneth J. Schafer, and Mette B. Gaarde. Multilevel perspective on high-order harmonic generation in solids. *Phys. Rev. A*, 94:063403, Dec 2016.

[34] F. Catoire, H. Bachau, Z. Wang, C. Blaga, P. Agostini, and L. F. DiMauro. Wannier representation of intraband high-order harmonic generation. *Phys. Rev. Lett.*, 121:143902, Oct 2018.

[35] P Jürgens, B Liewehr, B Kruse, C Peltz, D Engel, A Husakou, T Witting, M Ivanov, MJJ Vrakking, T Fennel, et al. Origin of strong-field-induced low-order harmonic generation in amorphous quartz. *Nature Physics*, 16(10):1035–1039, 2020.

[36] Giulio Vampa, TJ Hammond, Nicolas Thiré, BE Schmidt, François Légaré, CR McDonald, Thomas Brabec, DD Klug, and PB Corkum. All-optical reconstruction of crystal band structure. *Physical review letters*, 115(19):193603, 2015.

[37] G Vampa, CR McDonald, G Orlando, PB Corkum, and T Brabec. Semiclassical analysis of high harmonic generation in bulk crystals. *Physical Review B*, 91(6):064302, 2015.

[38] G Vampa, CR McDonald, G Orlando, DD Klug, PB Corkum, and T Brabec. Theoretical analysis of high-harmonic generation in solids. *Physical review letters*, 113(7):073901, 2014.

[39] Alexis Chacón, Dasol Kim, Wei Zhu, Shane P. Kelly, Alexandre Dauphin, Emilio Pisanty, Andrew S. Maxwell, Antonio Picón, Marcelo F. Ciappina, Dong Eon Kim, Christopher Ticknor, Avadh Saxena, and Maciej Lewenstein. Circular dichroism in high-order harmonic generation: Heralding topological phases and transitions in Chern insulators. *Phys. Rev. B*, 102(13):134115, October 2020. arXiv: 1807.01616.

[40] Lun Yue and Mette B. Gaarde. Imperfect Recollisions in High-Harmonic Generation in Solids. *Phys. Rev. Lett.*, 124(15):153204, April 2020.

[41] János K Asbóth, László Oroszlány, and András Pályi. A short course on topological insulators. *Lecture notes in physics*, 919:166, 2016.

[42] Di Xiao, Ming-Che Chang, and Qian Niu. Berry phase effects on electronic properties. *Reviews of modern physics*, 82(3):1959, 2010.

[43] Michael Victor Berry. Quantal phase factors accompanying adiabatic changes. *Proceedings of the Royal Society of London. A. Mathematical and Physical Sciences*, 392(1802):45–57, 1984.

[44] Tran Trung Luu and Hans Jakob Wörner. Measurement of the berry curvature of solids using high-harmonic spectroscopy. *Nature communications*, 9(1):1–6, 2018.

[45] Amnon Yacoby, Moty Heiblum, Diana Mahalu, and Hadas Shtrikman. Coherence and phase sensitive measurements in a quantum dot. *Physical review letters*, 74(20):4047, 1995.

[46] Yang Ji, Yunchul Chung, D Sprinzak, Moty Heiblum, Diana Mahalu, and Hadas Shtrikman. An electronic mach–zehnder interferometer. *Nature*, 422(6930):415–418, 2003.

[47] Yuval Ronen, Thomas Werkmeister, Danial Haie Najafabadi, Andrew T Pierce, Laurel E Anderson, Young Jae Shin, Si Young Lee, Young Hee Lee, Bobae Johnson, Kenji Watanabe, et al. Aharonov–bohm effect in graphene-based fabry–pérot quantum hall interferometers. *Nature nanotechnology*, 16(5):563–569, 2021.

[48] Shicheng Jiang, Hui Wei, Jigen Chen, Chao Yu, Ruifeng Lu, and C. D. Lin. Effect of transition dipole phase on high-order-harmonic generation in solid materials. *Phys. Rev. A*, 96(5):053850, November 2017.

[49] Shicheng Jiang, Chao Yu, Jigen Chen, Yanwei Huang, R. F. Lu, and C. D. Lin. How to obtain complex transition dipole moments satisfying crystal symmetry and periodicity from ab-initio calculations. *Phys. Rev. B*, 102(15):155201, October 2020. arXiv: 1912.09649.

[50] Dror Shafir, Hadas Soifer, Barry D Bruner, Michal Dagan, Yann Mairesse, Serguei Patchkovskii, Misha Yu Ivanov, Olga Smirnova, and Nirit Dudovich. Resolving the time when an electron exits a tunnelling barrier. *Nature*, 485(7398):343, 2012.

[51] Yann Mairesse, Nirit Dudovich, J Levesque, M Yu Ivanov, Paul B Corkum, and DM Villeneuve. Electron wavepacket control with elliptically polarized laser light in high harmonic generation from aligned molecules. *New Journal of physics*, 10(2):025015, 2008.

[52] Álvaro Jiménez-Galán, Rui EF Silva, Olga Smirnova, and Misha Ivanov. Sub-cycle valleytronics: control of valley polarization using few-cycle linearly polarized pulses. *Optica*, 8(3):277–280, 2021.
